# Autism-Related Information on Websites and General-Purpose Artificial Intelligence Chatbots: Comparative, Bilingual Study

**DOI:** 10.2196/85196

**Published:** 2026-07-13

**Authors:** Valentin Nădășan, Ciprian-Rareș Păroiu, Alexandra Neguțescu, Elena-Gabriela Strete

**Affiliations:** 1 Hygiene Department, George Emil Palade University of Medicine, Pharmacy, Science, and Technology of Targu Mures, Targu Mures, Mures County, Romania; 2 Psychiatry Clinic No 2, Mures County Clinical Hospital, George Emil Palade University of Medicine, Pharmacy, Science, and Technology of Targu Mures, Str. Gheorghe Marinescu, 38, Targu Mures, Mures County, 540142, Romania; 3 Faculty of Medicine, George Emil Palade University of Medicine, Pharmacy, Science, and Technology of Targu Mures, Targu Mures, Mures County, Romania; 4 Psychiatry Department, George Emil Palade University of Medicine, Pharmacy, Science, and Technology of Targu Mures, Targu Mures, Mures County, Romania

**Keywords:** autism spectrum disorder, online health information, chatbots, large language models, information quality, completeness and accuracy, multilingual assessment

## Abstract

**Background:**

Parents increasingly consult the internet, both websites and, more recently, artificial intelligence chatbots, for information on autism spectrum disorder (ASD). However, the comparative quality of these two source types, especially across languages, remains underexplored.

**Objective:**

This study aimed to assess the completeness and accuracy of ASD information delivered by websites and 5 popular artificial intelligence chatbots and determine whether performance differs between English and Romanian content.

**Methods:**

In a cross-sectional design, 25 English-language and 25 Romanian-language websites and the responses of ChatGPT, Gemini, Claude, Copilot, and DeepSeek were evaluated. Content was benchmarked against a 24-item checklist, yielding completeness and accuracy scores. Chatbots were tested in 2 scenarios: a single broad query (A) and 24 item-specific queries (B).

**Results:**

Websites achieved higher completeness in English than in Romanian (6.9 vs 5.1; *P*=.007) and marginally higher accuracy (6.9 vs 6.1; *P*=.045). In scenario A, chatbot completeness (English: 5.3 vs Romanian: 6.2; *P*=.15) and accuracy (English: 6.0 vs Romanian: 5.6; *P*=.32) did not show significant differences by language. In the single-query scenario, websites showed higher accuracy than chatbots in both English (6.9 vs 6.0; *P*=.19) and Romanian (6.1 vs 5.6; *P*=.53), with neither difference reaching statistical significance. Conversely, item-specific questioning favored chatbots, which yielded higher accuracy scores than websites in English (8.3 vs 6.9; *P*=.053) and Romanian (8.0 vs 6.1; *P*=.007). Accuracy scores improved significantly from the single-query to the item-specific scenario (English: 6.0 vs 8.3; *P*=.002; Romanian: 5.6 vs 8.0; *P*=.001). While an initial analysis suggested variation in performance between chatbots (repeated measures ANOVA; *P*=.005), pairwise differences between individual models did not remain significant after adjustment for multiple testing.

**Conclusions:**

This exploratory study indicates that the quality of online ASD information varies by language and source context. English-language websites are more complete than Romanian-language websites. Among chatbots, targeted questioning yields more accurate answers than single broad queries in both languages. The findings should be interpreted cautiously due to the temporal gap between website and chatbot data collection.

## Introduction

Autism spectrum disorder (ASD) is one of the most common neurodevelopmental disorders, with a steadily increasing prevalence over the past 2 decades [1]. This diagnosis significantly affects the quality of life of both patients and their caregivers or parents, requiring early detection and a complex, individualized treatment plan. The diagnosis often generates an emotional shock for the family and creates a highly uncertain context [2], leading to the need for additional, clear information and psychoeducational support. Collaboration between the family, professionals (physicians, therapists, psychologists), the educational system, and the social system is essential [3,4].

Consequently, it is natural for parents to seek information from the most accessible sources. As such, the majority of parents turn to the internet for this purpose [1,5,6]. This is partly due to the limited access to medical services, the short duration of specialist consultations, the potential lack of empathy from professionals, and the use of overly technical language that is not easily understood by the general public [1]. On the other hand, online information sources offer several advantages: accessibility, anonymity, and a variety of formats, including specialized websites, social networks, forums, mobile apps, and, in the last 2 to 3 years, artificial intelligence (AI)–based conversational agents, which are increasingly being used for this purpose, even though no official recommendations currently endorse their use [7,8].

The ease of access to this type of information carries a number of significant concerns, particularly regarding the quality of information available to parents, which remains insufficiently studied or regulated. As a result, parents may be exposed to inaccurate, harmful, or even dangerous content [9]. Equally troubling are the myths and misinformation propagated in this space, which can undermine parents’ trust in professionals and indirectly lead to delayed or incorrect diagnoses, with potentially devastating consequences throughout the lives of both the child and the family [10].

ChatGPT, along with other AI-based conversational models (large language models [LLMs]), represents one of the current challenges that has drawn attention from health care professionals as well [11]. The novel mode of interaction with these easily accessible models, capable of offering human-like answers to almost any question, has led to their rapid, widespread adoption. However, there is considerable uncertainty regarding the impact of this mass usage in the context of medical information. Assessments of ChatGPT’s performance have raised concerns about the confidence with which it provides responses, as it can deliver inaccurate answers with persuasive confidence [12]. Moreover, it has underperformed in tasks involving differential diagnosis and, in some cases, has offered potentially harmful treatment recommendations [8].

To date, few studies have assessed the credibility and quality of ASD-related information available on websites [13,14], and the gaps are even more significant concerning the evaluation of autism-related content provided by general-purpose AI conversational agents [1].

Our study aimed to investigate the quality of autism-related information available on 2 major categories of online sources: websites and general-use AI conversational agents. The primary objective was to assess the quality of ASD-related information provided by online sources, namely websites and chatbots. The secondary objective was to identify potential differences between various websites and chatbots, as well as between the languages in which the information was delivered.

## Methods

### Overview

This research was designed as an observational, cross-sectional study with 2 core components: the first focusing on the quality of autism-related information available on websites and the second on the quality of autism-related information provided by AI chatbot interfaces. The study is part of a broader project on the hygiene of medical information that aims to evaluate the quality of online information about the most common diseases and applies a standard methodology described in previously published work [14-16]. The essential elements of the methodology are described below.

### Study of Website Articles

The study was conducted from August to September 2019 and included websites that provide information on ASD. We selected English-language sites, the most widely used language globally, and Romanian-language sites, a language of local importance, to explore possible particularities of online information in a language with limited circulation. To identify the websites, we used the Google search engine, which is used by the vast majority of users. Although studies of user behavior show a clear preference for results in the first 5 to 10 positions, we included 25 websites for each language to capture less common search patterns, such as extending the search beyond the top 10 Google results [17]. Although this sampling approach was intended to capture a broader range of search results, no formal a priori power calculation was performed, and the study was not designed to be specifically powered for between-group comparisons.

Eligible sites were identified by entering the query term “autism” in English (on the global Google page) and in Romanian (on the local Romanian Google page). The term was chosen based on its popularity, as indicated by Google Trends, a tool that analyzes the frequency of top searches conducted in Google Search across regions and languages.

The search result pages were exported and archived as PDF files to document the search output at the time of data collection. The archived English-language and Romanian-language search results are provided in MultimediaAppendices 1  2, respectively. The results were screened sequentially according to their ranking on the Google results page, and the first 25 websites meeting the inclusion criteria were selected. To be included, a site had to address ASD, present information in the target language, and contain at least 300 words, with content intended for lay users without medical training. We excluded pages dealing with topics other than autism, sponsored results displayed at the top of the list, and pages that were compromised or inaccessible. We also excluded sites consisting exclusively of audio or video content and those requiring registration or payment for access. Pages that treated the topic only as news pieces or forum or social-media comments, rather than providing an in-depth explanation, were left out of the sample. Eligible sites were analyzed consecutively following a rigorous protocol.

After sampling, each text was evaluated for content quality against a 24-item checklist covering essential ASD concepts. The checklist was developed specifically for this study based on peer-reviewed literature and evidence-based guidance on ASD [18-22], with the aim of covering information considered both necessary and understandable for lay users. It included the following aspects: What is autism and how prevalent is it? What signs and symptoms should prompt suspicion? Which factors raise the risk of developing the condition? How does autism typically progress, and what complications may arise? What treatment options are available? The checklist was reviewed by 2 practicing adult psychiatrists to assess its relevance and comprehensibility for nonspecialist readers; however, it was not formally validated as an external measurement instrument. The full 24-item evaluation checklist used in this study is provided in Multimedia Appendix 3.

For each item, the presence (coded as 1) or absence (coded as 0) of the information was recorded; the sum represented the absolute completeness score (ACS), which was transformed into a relative completeness score (RCS) ranging from 0 to 10 using the formula:


RCS=10×ACS/24

The accuracy of each concept presented on the websites was rated on an ordinal scale from 0 to 2, where 0 indicated incorrect information with minimal or absent details, 1 indicated partially correct information with partial details, and 2 indicated correct information with complete details. The sum of item scores yielded the absolute accuracy score (AAS), which was transformed into a relative accuracy score (RAS) ranging from 0 to 10 using the formula:


RAS=10×AAS/MAS

where MAS represents the maximum accuracy score, which was a site-specific maximum accuracy score, the highest possible number of points that a specific site could be awarded, assuming all the items addressed were completely accurate.

Completeness and accuracy were evaluated as separate but complementary dimensions of information quality. The RAS was calculated only for the checklist items that were addressed by the source, reflecting the correctness of the information provided. In contrast, the RCS captured the proportion of checklist items addressed by the source. Consequently, a response that addressed only a small number of items could achieve a high accuracy score if the information provided was correct, but it would simultaneously receive a low completeness score, reflecting the limited scope of the information.

Completeness and accuracy were assessed by 2 independent raters following identical instructions. Interrater agreement was checked using Cohen κ test, with an acceptable agreement threshold of 0.8. For websites with κ <0.8, raters reexamined divergent scores and assigned a final score by discussion and consensus.

### Study of Chatbot-Generated Responses

The second component of the study was conducted between June and July 2025 and assessed the quality of ASD information provided in English and Romanian by 5 general-purpose conversational agents accessed through their publicly available web interfaces: ChatGPT-4o (OpenAI), Gemini 2.5 Flash (Google DeepMind), Claude Sonnet 4 (Anthropic), Microsoft Copilot (powered by GPT-4 Turbo), and DeepSeek-R1 (DeepSeek). All chatbot interactions were performed using the free versions available to the general public at the time of data collection, without paid subscriptions or premium access.

Selection criteria for AI chatbots were relevance and popularity among the general public (ChatGPT, Gemini, and Copilot are among the most widely used conversational interfaces worldwide, while Claude and DeepSeek are rapidly growing models); diversity of providers and ecosystems (including multiple major and independent companies to reduce the risk of bias associated with a single technological infrastructure); multiplatform accessibility (Windows and Apple operating systems) with web or mobile interfaces accessible to the public; functionality in multiple languages (English and Romanian); and free, globally available access. The number of chatbots included was determined pragmatically on the basis of these criteria and was not based on a formal a priori power calculation.

Each chatbot was queried under 2 complementary scenarios: in scenario A, a single broad question was asked (“What should a parent know about ASD?”), while scenario B used 1 specific question for each of the 24 items in the checklist described in the previous section. The full set of English-language prompts used in scenarios A and B is provided in Multimedia Appendix 4. The questions were drafted by 1 study author and reviewed by the other authors to simulate how a layperson without medical training would ask.

Each prompt was submitted once, and no repeated queries were performed. Due to usage limits associated with some free chatbot interfaces, certain prompts were completed across multiple sessions when necessary. Only the predefined study questions were submitted, and no additional prompts were used. Responses were generated using the default system settings, as generation parameters such as temperature are not user-adjustable in these public interfaces.

Quality indicators (completeness and accuracy) and the evaluation procedure were identical to those applied to websites to allow direct comparisons. The only notable difference was that the completeness assessment of chatbot responses was relevant only in scenario A, considering that in scenario B, where 1 question was posed for each checklist item, the completeness score would have been automatically and invariably 10. Worked examples showing how selected chatbot responses were mapped onto the checklist and scored are provided in Multimedia Appendix 5.

As with the websites, the 2 dimensions of information quality provided by chatbots were rated by 2 independent evaluators, who recorded the presence of items and judged accuracy according to the checklist. Interrater agreement was verified using Cohen κ, and discrepancies were resolved by the same consensus procedure.

The overall mean score for each chatbot was calculated as the arithmetic mean of all reported performance scores, namely scenario A completeness and accuracy scores in English and Romanian and scenario B accuracy scores in English and Romanian, with equal weighting of all components. The SD was calculated from the same set of values.

### Statistical Analysis

Completeness and accuracy scores were calculated using the formulas mentioned above. The normality of score distributions was tested using the Kolmogorov-Smirnov test. For 2-group comparisons with normal distributions, we used the independent or paired *t* test, as appropriate; when normality was not met, we used the Mann-Whitney *U* test. To compare the performance of the 5 individual chatbots on 6 quality-of-information indicators, we applied the one-way repeated-measures ANOVA (within-subjects factor=chatbot). Statistical significance was set at *α*=.05.

### Ethical Considerations

This study exclusively evaluated publicly available online text from websites and automated responses generated by general-purpose AI chatbots. The research did not involve human participants, clinical trials, or the collection, storage, or analysis of identifiable personal or medical data. The 24-item evaluation checklist was reviewed by 2 practicing adult psychiatrists solely to assess its clinical relevance and comprehensibility for nonspecialist readers, without any interaction with patients or patient records. Consequently, institutional review board approval and informed consent were not required for this study under national and institutional regulations.

## Results

**Figure 1.**
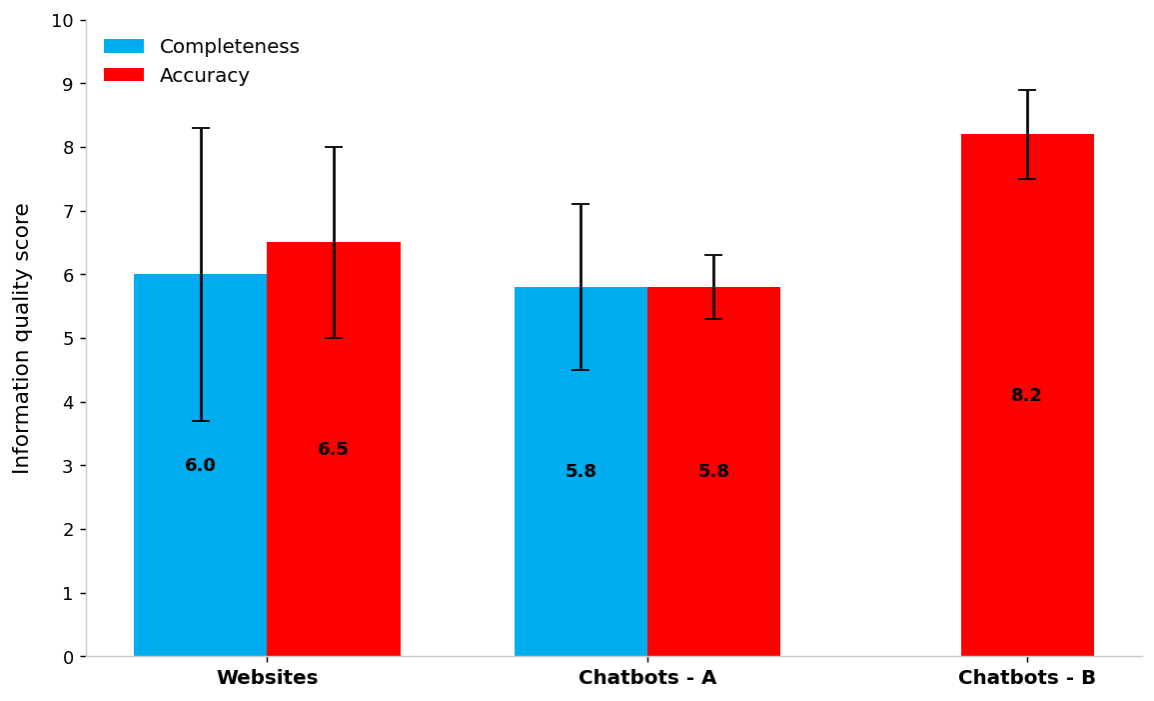
Overall completeness and accuracy scores for information about autism across websites and general-purpose chatbots. Scenario A: single, general question. Scenario B: multiple, specific questions. Error bars represent SDs.

Figure 1 displays the overall completeness and accuracy of the information about ASD available to general users from the investigated sources.

Table 1 summarizes and compares the completeness and accuracy of ASD information by language (English vs Romanian) across both traditional websites and generative AI chatbots, presenting descriptive statistics alongside the corresponding statistical test results. Table 2 shows the results of comparative tests aiming to discover information quality differences between websites and AI chatbots.

**Table 1.** Comparison of completeness and accuracy scores by language and information source (websites and chatbots).^a^

Source and scenario, and parameter	English, mean (SD)	Romanian, mean (SD)	*P* value
Websites			
Completeness	6.9 (2.3)	5.1 (2.2)	.007
Accuracy	6.9 (1.5)	6.1 (1.4)	.045
Chatbots (scenario A)			
Completeness	5.3 (1.3)	6.2 (1.3)	.15
Accuracy	6.0 (0.3)	5.6 (0.7)	.32
Chatbots (scenario B)			
Accuracy	8.3 (0.5)	8.0 (1.0)	.39

a Scores are expressed in points on a 0–10 scale (0=minimum, 10=maximum).

**Table 2.** Comparison of completeness and accuracy scores between websites and chatbots (scenarios A and B) by language.^a^

Language and parameters	Websites, mean (SD)	Chatbots, mean (SD)	*P* value
English			
Completeness	6.9 (2.3)	Scenario A: 5.3 (1.3)	.15
Accuracy	6.9 (1.5)	Scenario A: 6.0 (0.3)	.19
Accuracy	6.9 (1.5)	Scenario B: 8.3 (0.5)	.053
Romanian			
Completeness	5.1 (2.2)	Scenario A: 6.2 (1.3)	.31
Accuracy	6.1 (1.4)	Scenario A: 5.6 (0.7)	.53
Accuracy	6.1 (1.4)	Scenario B: 8.0 (1.0)	.007

a Scores are expressed in points on a 0–10 scale (0=minimum, 10=maximum).

For English, the 5 chatbots showed a mean accuracy of 6.0 (SD 0.3) in scenario A and 8.3 (SD 0.5) in scenario B (*P*=.002). For Romanian, the corresponding means were 5.6 (SD 0.7) in scenario A and 8.0 (SD 1.0) in scenario B (*P*=.001). Table 3 presents the completeness and accuracy scores obtained by each of the 5 chatbots, reported separately for English and Romanian queries in both test scenarios. The final row summarizes overall performance as the mean (SD) of 6 equally weighted scores for each chatbot: scenario A completeness (English, Romanian), scenario A accuracy (English, Romanian), and scenario B accuracy (English, Romanian).

**Table 3.** Completeness, accuracy, and combined performance scores of the 5 chatbots by language and scenario.^a^

Scenario and parameters or language	Gemini	ChatGPT	DeepSeek	Claude	Copilot
Scenario A					
Completeness-English	6.3	6.7	5.0	5.4	3.3
Completeness-Romanian	7.5	7.1	6.7	4.6	5.0
Accuracy-English	6.3	5.9	5.8	5.8	6.3
Accuracy-Romanian	6.4	5.6	6.3	5.0	5.0
Scenario B					
Accuracy-English	8.3	8.5	8.5	8.8	7.5
Accuracy-Romanian	9.0	7.9	8.5	8.3	6.5
Overall mean (SD)	7.3 (1.2)	7.0 (1.1)	6.8 (1.5)	6.3 (1.8)	5.6 (1.5)

a Scores are expressed in points on a 0–10 scale (0=minimum, 10=maximum). The 1-way repeated-measures ANOVA showed a *P* value of .005 in the within-subjects effects test. Pairwise comparisons showed Gemini and DeepSeek were superior to Copilot if unadjusted *P* values were considered (both *P*=.02). When Bonferroni correction for multiple comparisons was applied, differences between chatbots were no more statistically significant in pairwise comparisons.

## Discussion

### Principal Findings

This cross-sectional evaluation compared the completeness and accuracy of autism-related information retrieved from traditional websites and 5 general-purpose LLM chatbots, in both English and Romanian. Overall, ASD-related information on both English- and Romanian-language websites was moderately complete and accurate, while chatbot responses were broadly comparable overall, with more favorable accuracy results observed under more specific prompting. English-language websites offered more complete coverage than Romanian ones. The roughly 2-point gap in completeness between languages likely reflects several missing topic areas, potentially reducing the practical usefulness of the guidance for parents searching in a less-resourced language. Chatbots matched website accuracy in the single-query scenario. However, when prompted item by item, the conversational agents showed higher accuracy scores than the website sample, particularly in Romanian. However, these cross-source comparisons should be interpreted cautiously. Between-chatbot variability was modest; Gemini and DeepSeek showed higher scores than Copilot on unadjusted pairwise comparisons, but these differences did not remain significant after correction for multiple testing. These patterns suggest a changing information landscape highlighting the potential of conversational AI in multilanguage contexts, although its capacity to fully mitigate linguistic gaps remains unproven, while still showing variability in performance, particularly when prompted broadly.

Several factors likely explain the observed findings. Websites represent static, human-curated repositories; English sites benefit from larger editorial teams, wider peer-to-peer review, and more abundant source material, hence their higher completeness. Romanian sites probably rely on much smaller teams and budgets, leading to fewer topic sections and slower updates. Chatbots, by contrast, draw on vast multilingual corpora, and transformer models exhibit strong cross-lingual transfer [23]. However, their generative nature means that spontaneous responses must compress large knowledge bases into a short answer, which can omit checklist items [24]. When prompted item by item, the model can focus on a narrower retrieval bandwidth, boosting factual density and accuracy. Future studies could further explore how token limits or response truncation influence the informational content and usability of chatbot-generated health information. Our observation complements the work of other authors, who have shown that different prompts lead LLMs to generate responses with varying levels of quality [25-27].

Our findings on the quality of ASD-related online information are consistent with previous studies indicating that LLMs, such as GPT-4 and Gemini, generally produce information of moderate-to-good quality about autism and, more broadly, other mental health disorders [1,28]. Earlier assessments of website quality for ASD reported moderate reliability using the DISCERN tool (46.5 of a possible 80) compatible, although not closely comparable to our results, due to the different nature of the assessment methodology [13].

Our cross-lingual analysis, which found that English-language sources consistently scored higher than Romanian sites on both completeness and accuracy, mirrors and extends several earlier lines of evidence. First, Chu et al [29] showed that non-English speakers, particularly Chinese- and Spanish-language respondents, were more than twice as likely to report frustration, doubt the credibility, and have difficulty understanding online health information, underscoring a perceived quality gap linked to language preference. Second, the classic audit by Berland et al [30] demonstrated a concrete content deficit: only 22% of key clinical elements were adequately covered on Spanish pages compared with 45% on English pages, although accuracy per se was similar. Third, previous studies in various areas, more or less adjacent to psychiatry, suggest that general-purpose AI chatbots are already improving cross-language patient education and communication. In acute ENT settings, for example, ChatGPT has delivered guideline-concordant advice on sudden sensorineural hearing loss in both English and Spanish with similar clinical accuracy [31]. Within dermatology, board-certified clinicians have judged Spanish and Russian versions of patient-education materials generated by the same model to be clinically usable, showing progress toward language-concordant care even if performance for languages such as Mandarin remains uneven [32]. Likewise, GPT-4 has produced multilingual pediatric discharge instructions that faithfully preserve meaning and introduce markedly fewer critical errors than standard machine-translation services, demonstrating its capacity to support caregivers across linguistic boundaries [33].

A plausible explanation for these cross-linguistic differences is the unequal representation of languages in the data used to train LLMs. Higher completeness and accuracy scores in English responses may reflect the broader language-resource imbalance in LLM training. Most LLMs are trained predominantly on English-language corpora, whereas other languages are comparatively underrepresented, which may contribute to reduced performance in non-English contexts. Previous studies evaluating LLM performance across languages have reported similar disparities, with higher accuracy and content quality in English than in other languages. These findings suggest that multilingual chatbot responses may not simply represent direct translations of equivalent knowledge but may also reflect differences in the availability and representation of language-specific training data. This phenomenon highlights a potential digital language divide, with important implications for parents seeking health information in languages other than English [34].

Another relevant factor that was not directly assessed in this study is the readability of the provided information. Parents seeking information after an autism diagnosis often experience significant emotional stress and may benefit from clear, concise explanations rather than highly technical descriptions. Although this analysis focused on the completeness and accuracy of information, future research should also evaluate readability and cognitive accessibility using established metrics such as Flesch-Kincaid or similar readability indices [35].

Parents typically seek information online soon after suspecting ASD, often before a formal evaluation [1,2]. Incomplete or inaccurate content heightens the risk of delayed diagnosis, adoption of unproven therapies, or abandonment of evidence-based interventions [9,10]. Another aspect that may influence how parents perceive online information about autism is the psychological context in which information-seeking occurs. Parents searching for information after a suspected or confirmed diagnosis may be experiencing uncertainty, distress, and a need for reassurance. In this setting, the perceived usefulness of information may depend not only on factual accuracy but also on communicative features such as clarity, tone, and perceived empathy. This may be particularly relevant for chatbot-generated responses, as recent literature suggests that LLMs can exhibit elements of cognitive empathy and emotional support. Therefore, the attractiveness of these tools may partly reflect not only informational quality but also the interpersonal style in which information is delivered [36].

Our finding that Romanian websites scored the lowest on completeness underscores the vulnerability of families searching in a less-resourced language. On the other hand, the higher accuracy observed when parents asked chatbots specific questions suggests that AI tools may usefully supplement conventional resources, provided that users are able to formulate sufficiently granular prompts. Other prompt engineering techniques documented by various authors may further enhance the quality of AI chatbot answers. However, clinical teams, specifically pediatric doctors and nurses, may need to counsel caregivers on effective, checklist-style questioning while emphasizing cross-checking with professional advice [37-39].

First, there is a substantial temporal mismatch between the 2 data sources: websites were sampled in 2019, whereas chatbot responses were collected in 2025. Given the rapid evolution of the online health information ecosystem and the emergence of advanced LLMs during this 6-year gap, this temporal difference introduces a notable confounding factor. While some longitudinal evidence suggests that certain consumer-facing health website content may exhibit structural stability over several years [14], any direct statistical or conceptual comparisons between the 2019 website snapshot and the 2025 chatbot outputs must be interpreted with extreme caution and treated as strictly exploratory rather than definitive. Additionally, because search engine results vary over time, by location, and according to algorithm updates, the website data represent a point-in-time snapshot of the digital environment. Furthermore, the chatbot evaluation was based on a single submission for each prompt; given the stochastic nature of LLMs, responses can vary across sessions or system updates, and this study does not account for the longitudinal stability of the AI-generated outputs. Second, the checklist used to assess completeness and accuracy was specifically developed for this study and, although based on peer-reviewed literature and reviewed by 2 practicing adult psychiatrists, it was not formally validated as an external measurement instrument. Therefore, some degree of expert judgment and subjectivity cannot be fully excluded. Third, the scope of our language analysis was limited to English and Romanian. While this allowed for a focused comparison between a dominant global language and a less-resourced Central-Eastern European language, the findings may not necessarily extend to other low-resource or non–Indo-European languages. Finally, the assessment of information quality was conducted by clinicians, who evaluated completeness and accuracy from a professional standpoint. As a result, our analysis does not capture how well this information would be understood or perceived by lay users, such as parents or caregivers of autistic children, a perspective that remains essential for evaluating real-world accessibility and usefulness. Future research should focus on the formal validation of such scoring instruments through a multidisciplinary approach involving not only clinicians but also parents and autistic individuals themselves to ensure that information quality benchmarks align with the actual needs and cognitive load of the target audience.

Further investigation should build on these findings by addressing several important directions. A key next step would be to sample websites at the same time as chatbot queries, allowing for a more accurate comparison of the current digital information landscape and its evolution over time. Expanding the study to include additional languages, particularly low-resource ones, and applying culturally adapted evaluation tools would enhance the generalizability and relevance of results across diverse populations. It is also critical to involve lay stakeholders, such as parents and autistic adults, in the assessment process to better capture perceived trustworthiness, usability, and overall accessibility of the information. Additional work is needed to examine the performance of other LLMs and domain-specific medical chatbots, as well as their potential influence on real-world decision-making. Some conversational systems, such as Perplexity, integrate chatbot functionality with search engine capabilities and provide explicit source citations. Such features may increase perceived transparency and trustworthiness for some users. However, this study focused on widely used general-purpose conversational agents that are commonly accessed by the general public. An important next question is whether citation-supported systems influence how parents evaluate the credibility of online autism-related information. In this context, controlled trials comparing structured versus unstructured chatbot use could provide valuable insights into the clinical use of AI-guided information seeking, particularly in caregiving and support contexts.

Although this study did not apply a separate formal safety framework, a full-response review during scoring did not identify clinically dangerous recommendations, unsafe interventions, or clearly harmful hallucinations. Lower scores generally reflected incompleteness rather than overtly misleading content.

### Conclusions

Overall, autism-related information on English- and Romanian-language websites achieved moderate levels of completeness and accuracy. Within the scope of this evaluation, chatbot answers were broadly comparable and, when guided by detailed, topic-specific prompts, tended to show improved accuracy scores, within the context of the prompts used. However, given the temporal gap between website and chatbot data collection, alongside the limited sample size and inherent methodological limitations, this study should be viewed strictly as an exploratory assessment. Consequently, these data do not provide strong evidence that conversational AI can completely bridge existing language gaps in online health resources. Nevertheless, observed language gaps in website content suggest that English pages covered more checklist items than Romanian ones, potentially putting families who search in a low-resource language at a relative disadvantage.

These findings point to 2 complementary priorities: expanding high-quality web content in underserved languages and helping parents craft precise questions that may improve the quality of chatbot responses. Importantly, online resources, whether websites or AI tools, should complement, not replace, an early consultation with a qualified health care professional when autism is suspected. Addressing these needs may contribute to a health-information ecosystem that is safer, more equitable, and truly responsive to users.

## Supplementary material

10.2196/85196 Multimedia Appendix 1 PDF archive of the first 100 English Google search results for “autism” (April 29, 2019).

10.2196/85196 Multimedia Appendix 2 PDF archive of the first 100 Romanian Google search results for “autism” (April 29, 2019).

10.2196/85196 Multimedia Appendix 3 Autism spectrum disorder information quality checklist and scoring criteria (English and Romanian).

10.2196/85196 Multimedia Appendix 4 English-language prompts used for chatbot evaluation.

10.2196/85196 Multimedia Appendix 5 Illustrative examples of checklist-based scoring for chatbot responses.

## Abbreviations

AAS: absolute accuracy score
ACS: absolute completeness score
AI: artificial intelligence
ASD: autism spectrum disorder
LLM: large language model
MAS: maximum accuracy score
RAS: relative accuracy score
RCS: relative completeness score
