# Supplementary material for: Autism-Related Information on Websites and General-Purpose Artificial Intelligence Chatbots: Comparative, Bilingual Study
Source: JMIR Form Res. 2026 Jul 13;10:e85196. doi: 10.2196/85196 (PMC13361620; doi:10.2196/85196)
Supplement: Multimedia Appendix 1 [file formative-v10-e85196-s001.pdf]

Google

autism

All | Images | News | Videos | Maps | More | Settings | Tools

About 242,000,000 results (0.68 seconds)

Autism is a developmental disorder characterized by difficulties with social interaction and communication, and by restricted and repetitive behavior. Parents usually notice signs during the first three years of their child's life.

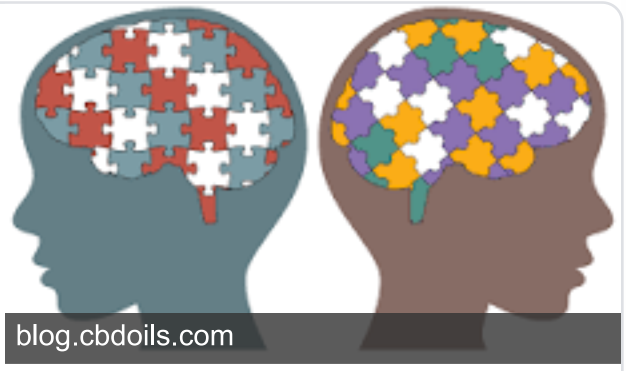

Symptoms: Trouble with social interaction, imp...

Differential diagnosis: Reactive attachment dis...

Diagnostic method: Based on behavior and de...

Complications: Social isolation, employment p...

Autism - Wikipedia

https://en.wikipedia.org › wiki › Autism

About Featured Snippets   Feedback

People also ask

What are the 3 main symptoms of autism?

What are the 5 different types of autism?

What causes autism?

What exactly is autism?

Feedback

Autism - Wikipedia

https://en.wikipedia.org › wiki › Autism ▾

Autism is a developmental disorder characterized by difficulties with social interaction and communication, and by restricted and repetitive behavior. Parents usually notice signs during the first three years of their child's life.

Causes: Genetic and environmental factors

Medication: Antipsychotics, antidepressants, st...

Differential diagnosis: Reactive attachment dis...

Treatment: Behavioral therapy, speech therapy, ...

Autism spectrum · Causes of autism · Regressive autism · Heritability of autism

What Is Autism? | Autism Speaks

https://www.autismspeaks.org › what-autism ▾

Autism, or **autism** spectrum disorder (ASD), refers to a broad range of conditions characterized by challenges with social skills, repetitive behaviors, speech and nonverbal communication. ... * In 2013, the American Psychiatric Association merged four distinct **autism** diagnoses into one ...

Top stories

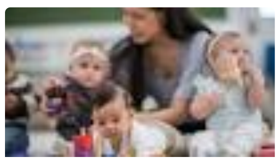

Infants should be tested for autism if their siblings are diagnosed, study suggests

Global News · 16 hours ago

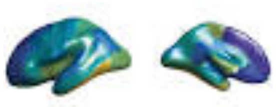

Brains of autistic people show unusual left-right symmetry

Spectrum | Autism Research · 1 day ago

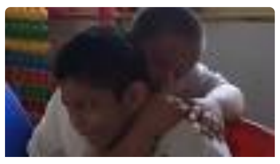

Little boy with Down's syndrome comforts his autistic classmate with a HUGE hug

Daily Mail · 11 hours ago

→ More for autism

Autism: Definition, Symptoms, Causes, & Types - WebMD

https://www.webmd.com › Brain & Nervous System › Autism ▾

Oct 22, 2019 - **Autism**, also called **autism** spectrum disorder (ASD), is a complicated condition that includes problems with communication and behavior. ... **Autism** Spectrum Disorders. ... Now, they fall under the range of **autism** spectrum disorders.

NIMH » Autism Spectrum Disorder

https://www.nimh.nih.gov › health › topics › autism-spectrum-disorders-asd ▾

Autism spectrum disorder (ASD) is a developmental disorder that affects communication and behavior. Although **autism** can be diagnosed at any age, it is said to ...

Autism spectrum disorder - Symptoms and causes - Mayo Clinic

https://www.mayoclinic.org › symptoms-causes › syc-20352928 ▾

Jan 6, 2018 - **Autism** spectrum disorder is a serious condition related to brain development that impairs the ability to communicate and interact with others.

What is Autism Spectrum Disorder? | CDC

https://www.cdc.gov › ncbddd › autism › facts ▾

Autism Spectrum Disorders (ASDs) are a group of developmental disabilities that can cause significant social, communication and behavioral challenges.

Videos

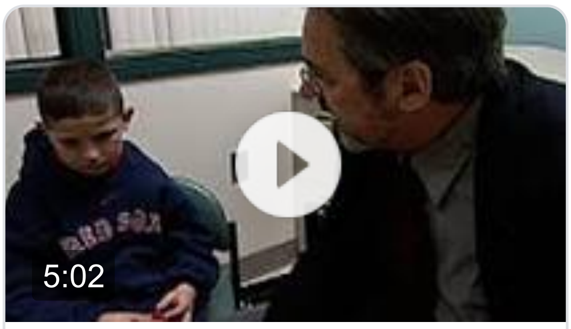

Inside an Autism Assessment

WebMD - Jun 2, 2017

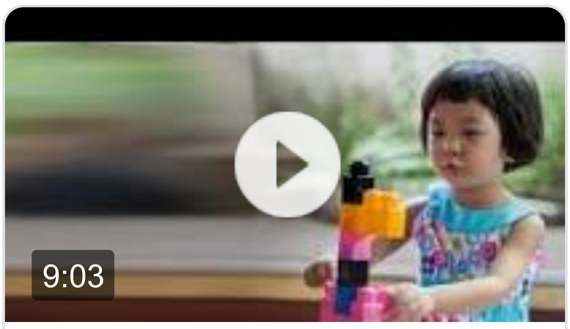

Early Signs of Autism Video Tutorial | Kennedy Krieger Institute

Kennedy Krieger Institute

YouTube - Jun 11, 2013

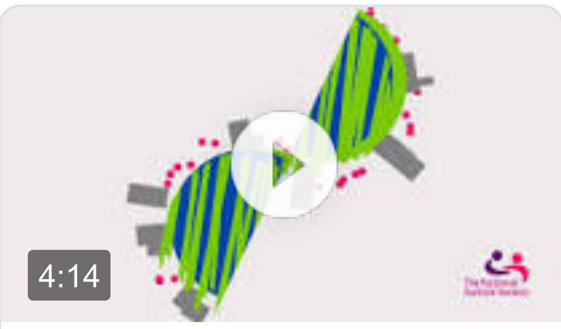

What is autism?

The National Autistic

YouTube - Apr 2, 2014

What is Autism? - Autism Society

https://www.autism-society.org › what-is ▾

Autism spectrum disorder is a complex developmental disability. **Autism** is defined by a certain

set of behaviors. There is no known single cause of **autism**.

Autism: Symptoms, Signs, Causes & Treatment - MedicineNet
https://www.medicinenet.com › autism_symptoms_and_signs › symptoms ▾
Learn the symptoms and signs of **autism**. Common symptoms and signs include behavioral disturbances, repetitive movements, and self-abusive behaviors.

Autism: Characteristics, diagnosis, and understanding
https://www.medicalnewstoday.com › articles ▾
Nov 20, 2018 - **Autism** is a set of psychological behaviors often characterized by an emphasis on routine and repetition, fixed behavioral patterns, and ...

Autism Spectrum Disorder: MedlinePlus
https://medlineplus.gov › Health Topics ▾
Oct 2, 2019 - Find out about **autism** spectrum disorder symptoms, causes, treatment, and other issues related to this developmental disorder.

Autism Spectrum Disorders - HelpGuide.org
https://www.helpguide.org › articles › autism-learning-disabilities › autism-... ▾
Nov 18, 2019 - An easy-to-understand guide to **autism** spectrum disorders, including common symptoms.

What Is Autism? Symptoms, Causes, Tests, Treatment, and More
https://www.healthline.com › health › autism ▾
Sep 18, 2018 - **Autism** spectrum disorders (ASDs) are neurological conditions that affect a person's ability to communicate with others. Get facts and statistics ...

Autism - NHS
https://www.nhs.uk › conditions › autism ▾
NHS guide to **autism**. Find out what **autism** is and what the common signs are. Get help and advice if you or your child are **autistic**.

Autism - National Autistic Society - autism.org.uk
https://www.autism.org.uk › about › what-is › asd ▾
Nov 15, 2019 - **Autism** is a lifelong developmental disability that affects how people perceive the world and interact with others. **Autistic** people can have ...

Autism spectrum disorders - World Health Organization
https://www.who.int › Newsroom › Fact sheets › Detail ▾
Nov 7, 2019 - **Autism** spectrum disorders consist of a range of conditions characterised by some degree of impaired social behaviour, communication and ...

What Is Autism - Understood.org
https://www.understood.org › getting-started › what-you-need-to-know ▾
When kids struggle in school or in life, their challenges don't always fit into a neat box. One example of this is **autism** spectrum disorder (or ASD). Kids with ...

Autistic - Autism | Psychology Today
https://www.psychologytoday.com › intl › basics › autism ▾
Autism is a developmental disorder that affects information processing in multiple ways. People with **autism** have difficulties with social and communication skills.

Autism Spectrum Disorder (for Kids) - Nemours KidsHealth
https://kidshealth.org › kids › autism ▾
Autism spectrum disorder makes it hard for kids to learn and communicate. Find out more in this

article for kids.

Autism and Autism Spectrum Disorders
https://www.apa.org › topics › autism ▾
Autism spectrum disorder (ASD) refers to a neurodevelopment disorder that is characterized by difficulties with social communication and social interaction and ...

Autism | HHS.gov
https://www.hhs.gov › programs › topic-sites › autism ▾
Autism Spectrum Disorder (ASD) represents a broad group of developmental disorders.

Autism - HealthyChildren.org
https://www.healthychildren.org › English › conditions › Autism › Pages ▾
autism~The American Academy of Pediatrics (AAP) recommends that all children be screened for ASD at their 18- and 24-month well-child checkups. Here are a ...

Autism: SAGE Journals
https://journals.sagepub.com › home › aut
Autism is a major, peer-reviewed, international journal, published 8 times a year, publishing research of direct and practical relevance to help improve the quality ...

Society (required): The Int Society for Research ...

Autism Spectrum Disorder Basics | Child Mind Institute
https://childmind.org › guide › guide-to-autism-spectrum-disorder ▾
Autism spectrum disorder (ASD) is a developmental disorder that is marked by two unusual kinds of behaviors: deficits in communication and social interaction, ...

Autism | Brain & Behavior Research Foundation
https://www.bbrfoundation.org › research › autism ▾
The Brain & Behavior Research Foundation has awarded more than $14 million to **Autism** Research since 1987.

What Is Autism Spectrum Disorder?
https://www.psychiatry.org › patients-families › what-is-autism-spectrum-d... ▾
Autism Spectrum Disorder is a complex developmental disorder that can cause problems with thinking, feeling, language and the ability to relate to others. Learn ...

Autism, Autistic Spectrum Disorders (ASD) and Pervasive ...
www.med.umich.edu › yourchild › topics › autism ▾
What are the **autistic** spectrum disorders (ASD)? **Autism** spectrum disorders, or ASD, are also called pervasive developmental disorders (PDD) because they ...

What is autism? – Autism Spectrum Australia (Aspect)
https://www.autismspectrum.org.au › About autism ▾
1 in 70 Australians are diagnosed with **autism** spectrum disorder. The word spectrum reflects the wide range of difference that people on the spectrum ...

What is Autism? - Autism Speaks Canada
https://www.autismspeaks.ca › What is Autism? › About Us ▾
What is **Autism** : The following information is not meant to diagnose or treat and should not take the place of personal consultation, as appropriate, with.

Rethinking repetitive behaviors in autism | Spectrum | Autism ...
https://www.spectrumnews.org › features › deep-dive › rethinking-repetiti... ▾

Nov 25, 2019 - **Autistic** people have long maintained that repetitive behaviors are beneficial. Emerging evidence in support of this idea is shaping new ...

What Is Autism Spectrum Disorder? - ScienceAlert

https://www.sciencealert.com › autism-spectrum-disorder ▾

Autism Spectrum Disorder (ASD) describes a range of complex neurological conditions that affect an individual's social, communication, and motor skills.

Autism (Autism Spectrum Disorder) - ASHA

https://www.asha.org › public › speech › disorders › Autism

Autism is also known as **Autism** Spectrum Disorder (ASD). People with **autism** have challenges with communication and social skills. They also have repetitive ...

Autism | Society | The Guardian

https://www.theguardian.com › society › autism ▾

MPs call for law change to reduce detention of young **autistic** people. Published: 1 ... New Zealand supermarket launches 'quiet hours' for customers with **autism**.

Autism Spectrum Disorder: Practice Essentials, Background ...

https://emedicine.medscape.com › article › 912781-overview ▾

Sep 30, 2019 - **Autism** spectrum disorder (ASD) manifests in early childhood and is characterized by qualitative abnormalities in social interactions, markedly ...

Autism spectrum disorder (ASD) | KidsHealth NZ

https://www.kidshealth.org.nz › autism-spectrum-disorder-asd ▾

Autism spectrum disorder (ASD) is a developmental disorder that affects communication, social skills and behaviour. If your child does have ASD, there are ...

autism | Definition, Symptoms, Neuropathology, & Diagnosis ...

https://www.britannica.com › science › autism ▾

Autism, developmental disorder affecting physical, social, and language skills, with an onset of signs and symptoms typically before age three. Classic **autism** is ...

Autism Europe

https://www.autismeurope.org ▾

Autism-Europe (AE) is an international association whose main objective is to advance the rights of **autistic** people and to help them improve their quality of life.

Autism - HuffPost

https://www.huffpost.com › life › topic › autism ▾

Autism news and opinion. ... She Didn't Know Her **Autistic** Son Could Be Tasered At School. Rosie Phillips says she found her nonverbal son dazed and with ...

Autism: Scientists discover why gene mutation leads to disorder

https://www.usatoday.com › story › news › nation › 2019/12/05 › autism-s... ▾

16 hours ago - Scientists at Northwestern University have discovered how a gene mutation plays a key role in developmental disabilities like **autism**.

Autism Spectrum Disorder | Kennedy Krieger Institute

https://www.kennedykrieger.org › Patient Care › Conditions ▾

Autism Spectrum Disorder (ASD) – is a brain-based developmental disability that affects a child's ability to communicate, understand language, play and relate ...

About Autism | NHGRI

https://www.genome.gov › Genetic-Disorders › Autism ▾

May 19, 2019 - **Autism** is a group of developmental disorders characterized by impaired social interactions, problems with verbal and nonverbal ...

Autism Awareness Australia: Homepage

https://www.autismawareness.com.au ▾

Autism Awareness Australia - Australia's leading voice for **autism**. Our goal is simple: To improve the lives of all Australians on the **autism** spectrum and the ...

Autism: Symptoms, Diagnosis and Treatment | Live Science

https://www.livescience.com › 34704-autism-symptoms-diagnosis-and-trea... ▾

May 12, 2016 - **Autism** spectrum disorder affects a person's ability to communicate, interact with others and behave appropriately in social situations.

What is Autism and Autism Spectrum Disorder (ASD ...

https://www.altogetherautism.org.nz › Diagnosis ▾

Autism is a lifelong neurodevelopmental condition that affects how people perceive the world, how they think and behave, and how they communicate and ...

Autism Spectrum Disorder | The Mighty

https://themighty.com › topic › autism ▾

Our community is made up of **autistic** voices and the people who support them.

Autism

https://autism.bandcamp.com ▾

Autism AUTISM is an instrumental Post Rock / Post metal band from Vilnius, Lithuania. Have you found peace?, released 04 February 2019 1. Rememorari 2.

Autism Canada – See the Spectrum Differently

https://autismcanada.org ▾

Do you suspect someone you love or yourself may have **autism**? ... A showcase for a wide range of creative work produced by people living with **autism**.

Autism | NAMI: National Alliance on Mental Illness

https://www.nami.org › learn-more › mental-health-conditions › autism ▾

Autism spectrum disorder (ASD) is a developmental condition that affect a person's ability to socialize and communicate with others. People with ASD can also ...

Research Autism | Autism Treatments| Autism Therapies ...

www.researchautism.net ▾

The Research **Autism** information service is part of the National **Autistic** Society and provides research-based information about **autism**, treatments and related ...

Autism Spectrum Quotient - Psychology Tools

https://psychology-tools.com › test › autism-spectrum-quotient ▾

Psychological test measuring your **Autism** Spectrum Quotient, also know as AQ.

What is Autism? - Autism Resource Centre (Singapore)

https://www.autism.org.sg › Living with Autism ▾

Aug 22, 2019 - **Autism** is a lifelong developmental disability that affects a person's ability to make sense of the world and relate with others. **Autism** comes from ...

Autism - The Lancet

https://www.thelancet.com › clinical › diseases › autism

by MD Shen - Cited by 9 - Related articles

Autism spectrum disorder is a term used to describe a constellation of early- appearing social

communication deficits and repetitive sensory–motor behaviours ...

Autism | Definition of Autism by Merriam-Webster
https://www.merriam-webster.com › dictionary › autism
Autism definition is - a variable developmental disorder that appears by age three and is characterized especially by difficulties in forming and maintaining social ...

My Childhood Autism Went Undetected. It Cost Me Part of My ...
https://undark.org › 2019/11/28 › early-detection-autism
Nov 28, 2019 - Opinion: There is little question among psychologists about the benefits of early detection of **autism**. Yet many children still slip through the ...

Autisme
https://www.autisme.com › autism › what-is-autism
Autism Spectrum Disorder (ASD) is a neurobiological development disorder, which manifests during the first three years of life and lasts throughout their lifetime.

Autism: Symptoms, Causes, Diagnosis, Treatment
https://www.verywellhealth.com › autism-overview-4014759
Nov 4, 2019 - **Autism** is a developmental disorder that starts in early childhood. Learn the signs, causes, diagnostic tests, and how to choose the right ...

What is Autism? - News Medical
https://www.news-medical.net › health › What-is-Autism
Jun 5, 2019 - **Autism** is not a single disease entity. It is part of a range of developmental disorders known as **autistic** spectrum disorders (ASD). They begin in ...

autism - latest news, breaking stories and comment - The ...
https://www.independent.co.uk › topic › autism
All the latest breaking news on **autism**. Browse The Independent's complete collection of articles and commentary on **autism**.

Autism | Psychiatric Times
https://www.psychiatrictimes.com › autism
Neurodevelopment Risk and the **Autism** Spectrum. An update on the major findings on the biology of ASDs and advances in diagnostic and interventional ...

Autism: More Than Meets the Eye - Scientific American Blog ...
https://blogs.scientificamerican.com › beautiful-minds › autism-more-than-...
Jun 19, 2019 - **Autism** spectrum disorder (ASD) is a wide spectrum, ranging from those with severe disabilities to highly functioning **autism**. Also, there are ...

What is Autism and Asperger's Syndrome? | Mencap
https://www.mencap.org.uk › learning-disability-explained › conditions
Autism and Asperger syndrome affect everyone differently, depending where they are on the spectrum. The signs may often be different, but communication, ...

World Autism Awareness Day 2 April - the United Nations
https://www.un.org › events › autismday
"On World **Autism** Awareness Day, we speak out against discrimination, celebrate the diversity of our global community and strengthen our commitment to the ...

autism research centre
https://www.autismresearchcentre.com
Research into the biomedical causes of **autism** spectrum conditions.

About Autism | Organization for Autism Research
https://researchautism.org › how-we-help › families › about-autism
Autism is a neurodevelopmental disorder that affects one's ability to communicate and interact socially. It is described as a spectrum disorder, which means that ...

Autism Ontario: Home
https://www.autismontario.com
Autism Ontario is the province's leading source of information and referral on **autism** and one of the largest collective voices representing the **autism** community.

Autism - RationalWiki
https://rationalwiki.org › wiki › Autism
5 days ago - **Autism**, also known as **autism** spectrum disorder, is an inborn, lifelong developmental disability that impacts language, communication and ...

Autism Fast Facts - CNN - CNN.com
https://edition.cnn.com › 2013/10/14 › health › autism-fast-facts
Jul 4, 2019 - Read CNN's **Autism** Fast Facts and learn more about **autism** and **autism** spectrum disorders.

autism - Wiktionary
https://en.wiktionary.org › wiki › autism
(the specific, instead of the generalized range): **autism** (the generalized range, instead of the specific); **autism** spectrum · **autistic** spectrum · **autism** spectrum ...

Autism spectrum disorder | NHS inform
https://www.nhsinform.scot › autistic-spectrum-disorder-asd
Aug 9, 2019 - **Autism** spectrum disorder (ASD) is a condition that affects social interaction, communication, interests and behaviour. Learn about ASD ...

What is Autism? - Autism Science Foundation
https://autismsciencefoundation.org › what-is-autism
When people refer to "**Autism**" today, they are usually talking about **Autism** Spectrum Disorders (ASD), which is a brain-based disorder characterized by ...

What Is Autism - Irish Society for Autism
https://autism.ie › Information › FAQ
Autism is a lifelong developmental disability that affects the way a person communicates and relates to people around them.

Autism - GaDOE
https://www.gadoe.org › Special-Education-Services › Pages › Autism
Autism is a developmental disability, generally evident before age three, that adversely affects a student's educational performance and significantly affects ...

About Autism | Autistic Self Advocacy Network
https://autisticadvocacy.org › about-asan › about-autism
Autism is a neurological variation that occurs in about one percent of the population and is classified as a developmental disability. Although it may be more ...

Is Autism an "Epidemic" or Are We Just Noticing More People ...
https://www.discovermagazine.com › health › is-autism-an-epidemic-or-ar...
Jul 11, 2012 - She is science editor at the Thinking Person's Guide to **Autism** and author of The Complete Idiot's Guide to College Biology. autism1.

Autistic spectrum disorders (ASD) | Mental Health Foundation

https://www.mentalhealth.org.uk › learning-disabilities › a-to-z › autistic-s... ▾

Autistic spectrum disorder (ASD) is a term used to describe a number of symptoms and behaviours which affect the way in which a group of people understand ...

Are autistic individuals the best workers around? - BBC Worklife

https://www.bbc.com › worklife › article › 20160106-model-employee-are... ▾

Jan 6, 2016 - Then you might want to follow the lead of other businesses that have begun to actively recruit **autistic** employees. "They're loyal and diligent ...

Autism spectrum disorder (autism) - Better Health Channel

https://www.betterhealth.vic.gov.au › health › conditionsandtreatments › a... ▾

Jul 31, 2013 - **Autism** spectrum disorder includes **autistic** disorder, Asperger's syndrome and pervasive developmental disorder not otherwise specified ...

Autism Fact Sheet | National Autism Association

https://nationalautismassociation.org › resources › autism-fact-sheet ▾

What is **Autism**? **Autism** is a bio-neurological developmental disability that generally appears before the age of 3; **Autism** impacts the normal development of the ...

Autism / Autistic Spectrum Disorders | National Council for ...

https://www.sess.ie › categories › autism-autistic-spectrum-disorders ▾

Autism Spectrum Disorder(ASD) is a neurological, developmental disorder which effects how people with ASD communicate, socialise and interact with others.

Autism Spectrum - an overview | ScienceDirect Topics

https://www.sciencedirect.com › topics › medicine-and-dentistry › autism-sp...

Autism Spectrum Conditions (ASC) are strongly biased towards males, with a male:female ratio of 4:1 for classic **autism** and over 10:1 for Asperger Syndrome.

Autism in Ontario | Ontario.ca

https://www.ontario.ca › page › autism-ontario ▾

Get updates about **autism** and the Ontario **Autism** Program. Find resources, supports, services and programs for families with children and youth with **autism**.

Neuroscience For Kids - Autism - University of Washington

https://faculty.washington.edu › chudler › aut ▾

Fred was diagnosed with **autism**, a neurological disorder that disrupts normal development. Some children with **autism** can attend school with children their own ...

Inclusive Hiring at Microsoft

https://www.microsoft.com › diversity › inside-microsoft › cross-disability ▾

Recruit, onboard, and development of individuals on the **Autism** spectrum. Learn more ... To learn more about the **Autism** Hiring Program, check out our FAQs.

The Autism Research Institute | #1 Advocate for Autism ...

https://www.autism.org ▾

ARI works to advance the understanding of **autism** by funding research and facilitating education on its causes and the potential treatments.

Autism New Zealand

https://www.autismnz.org.nz ▾

Autism New Zealand empowers people living with **autism** to make informed choices and take action: every step together.

Autism NJ
https://www.autismnj.org ▾
Together, we help those with **autism** connect to necessary resources and ... Building communities that embrace and accept people and families with **autism**.

Autism News -- ScienceDaily
https://www.sciencedaily.com › news › mind_brain › autism ▾
Autism symptoms and new approaches to treatment. Read current research on **autism** including early diagnosis of **autism** spectrum disorders, genetic factors ...

Ce este autismul? Tulburarea care afectează ... - Help Autism
https://www.helpautism.ro › autism › despre-autism ▾ Translate this page
Statisticile internaţionale ne arată că 1 din 68 de copii suferă de **autism**, iar datele înregistrate în ultimii ani în România indică o creştere continuă a numărului ...

Autism Spectrum Disorders (ASD) Resource Information: UC ...
https://health.ucdavis.edu › mindinstitute › resources › autism ▾
Information, support, and resources for **Autism** in our community and nationally.

Autism Signs in Children: What Is Autism Spectrum Disorder?
https://www.onhealth.com › content › autism_spectrum_disorder ▾
Jul 19, 2016 - **Autism** is a wide range or spectrum of brain disorders that is usually noticed in young children. **Autism** is also referred to as **Autism** Spectrum ...

Autism Association of Western Australia: Making a positive ...
https://www.autism.org.au ▾
Established in 1967, we're the largest specialist lifespan organisation in Australia providing services to people with **Autism**. The programs we develop are ...

What Is Autism – Autism Navigator
https://autismnavigator.com › what-is-autism ▾
The signs of **autism** can be detected by 18 to 24 months. And yet most children with **autism** are not diagnosed until at least 5 years of age and miss the ...

Autism Research News - Neuroscience News
https://neurosciencenews.com › neuroscience-topics › autism ▾
Autism. Research articles covering work related to austism spectrum disorders are provided below. This shows a DNA double helix ...

Autism | News, Videos & Articles - Global News
https://globalnews.ca › tag › autism ▾
Autism videos and latest news articles; GlobalNews.ca your source for the latest news on **Autism** .

OMIM Entry - % 209850 - AUTISM
https://www.omim.org › entry ▾
Autism, the prototypic pervasive developmental disorder (PDD), is usually apparent by 3 years of age. It is characterized by a triad of limited or absent verbal ...

Using Technology to Close the Autism Job Gap - The New ...
https://www.nytimes.com › 2019/10/24 › business › autism-jobs-daivergent
Oct 24, 2019 - Adults on the **autism** spectrum often have difficulty finding employment. Entrepreneurs and advocates are working to fix that by pairing ...

Introduction to Autism | Interactive Autism Network

https://iancommunity.org › introduction-autism

Autism spectrum disorder, the name adopted in 2013, is a developmental disorder characterized by persistent problems in social communication and interaction, ...

The autism spectrum | TED Talks

https://www.ted.com › playlists › the_autism_spectrum

Explore the science of **autism** -- and listen to the stories of people who live with it.

Center for Autism | Cleveland Clinic Children's

https://my.clevelandclinic.org › pediatrics › departments › autism

Cleveland Clinic Children's Center for **Autism** is dedicated to the treatment, education, and research for children, adolescents and young adults with **autism** ...

Searches related to autism

autism **causes**	autism **in adults**
autism **wiki**	autism **treatment**
autism **symptoms**	autism **spectrum**
**types of** autism	autism **test**
