# Supplementary material for: Autism-Related Information on Websites and General-Purpose Artificial Intelligence Chatbots: Comparative, Bilingual Study
Source: JMIR Form Res. 2026 Jul 13;10:e85196. doi: 10.2196/85196 (PMC13361620; doi:10.2196/85196)
Supplement: Multimedia Appendix 2 [file formative-v10-e85196-s002.pdf]

Google

autismul

Conectați-vă

Toate Imagini Videoclipuri Știri Hărți Mai multe Setări Instrumente

Aproximativ 332.000 rezultate (0,36 secunde)

Ce este autismul? Tulburarea care afectează peste 30.000 de copii ...
https://www.helpautism.ro/autism/despre-autism
Autismul este o afecţiune considerată drept una dintre cele mai frecvente tulburări ale copilăriei, mai frecventă decât cancerul, diabetul şi sindromul Down.

Autism - Wikipedia
https://ro.wikipedia.org/wiki/Autism
Autismul este o tulburare de dezvoltare considerată drept una dintre cele mai severe tulburări neuropsihiatrice ale copilăriei. **Autismul** este tulburarea centrală ...

ICD-10: F84.0

Simptome · Cauze · Terapie · Note

Ce este autismul? - Asociatia pentru Interventie Terapeutica in Autism
https://www.autism-aita.ro/ce-este-autismul
Autismul inregistreaza o crestere alarmanta in toate mediile, in toata lumea, estimandu-se ca in anul 2012 exista 1 copil cu tulburare de spectru **autist** la 100 de ...

Zece semne de alarma pentru autism
https://www.autism-aita.ro/zece-semne-de-alarma-autism
Aici ajutam copiii sa fie din ce in ce mai putin **autisti**, mai fericiti si mai independenti. AITA se adreseaza copiilor intre 2 si 6 ani. Cele mai noi pagini din AITA.

Ce este autismul și care sunt simptomele. Când apare și cum se ...
https://www.libertatea.ro › Lifestyle › Sănătate și Fitness
16 feb. 2018 - **Autismul** este o tulburare de dezvoltare considerată drept una dintre cele mai severe tulburări neuropsihiatrice. Citește mai multe.

Perspective - „Autismul nu este o boala, ci o stare continua" | Secom.ro
https://www.secom.ro/perspective/autismul-nu-este-o-boala-ci-o-stare-continua
6 oct. 2017 - La ora actuala, se considera ca **autismul** nu este o boala, ci o stare bioneurologica. Copilul se naste cu anumite particularitati biologice care se ...

Semnele autismului | Medlife
https://www.medlife.ro/semnele-autismului.html
9 aug. 2013 - **Autismul** este o tulburare a creierului care limiteaza capacitatea unei persoane de a comunica si de a relationa cu alte persoane.Afectiunea ...

Autism infantil - Dr. Victor Babes
www.cdt-babes.ro/articole/autism-infantil.php
Autismul infantil, alaturi de sindromul Asperger si **autismul** atipic, face parte din Tulburarile pervazive de dezvoltare.

Autismul - Sfatul medicului
www.sfatulmedicului.ro/Autismul/autismul_14
21 nov. 2018 - **Autismul** - **Autismul** este o tulburare de dezvoltare a creierului care interfera adesea cu abilitatea de a comunica si de a relationa cu cei din jur.

Ce este autismul: cauze, diagnostic, tratament - Doc.ro
https://doc.ro/sanatate/ce-este-autismul-cauze-diagnostic-tratament
14 feb. 2017 - **Autismul** este o tulburare severa de dezvoltare, de natura neuro-biologica, in care persoanele afectate au dificultati in a dezvolta relatii sociale ...

Videoclipuri

6:08
Transcript of "Cum m-a eliberat autismul să devin cine sunt"
TED.com - 21 nov. 2014

5:07
Mihnea. Ce este autismul?
Valentin Tigau
YouTube - 26 nov. 2014

52:43
Ce este și mai ales ce NU este autismul
Canal 33
YouTube - 17 apr. 2018

[PDF] 1 Autismul Cuprins: • Ce este autismul? • Autismul si Muzica • Mituri ...
www.musicastherapy.org/uploads/R-_Autism_revised_Aug_16.doc.pdf
Autismul este o dizabilitate permanenta de dezvoltare care implica un defect organic in functionarea creierului. El afecteaza copiii si adultii din punct de vedere ...

Ce este și cum se manifestă autismul la copiii de 0-3 ani - Atlashelp.net
https://atlashelp.net › Acasă › Blog
3 mai 2018 - **Autismul** este una dintre tulburările neuropsihiatrice ale copilăriei, cu simptome precum dificultatea de a socializa și de a comunica a copilului.

Autismul NU este o boala! - CSID
https://www.csid.ro › Health › Sănătate
Autismul a constituit dintotdeauna un subiect controversat. In ani '50, atat societatea, cat si comunitatea medicala invinuiau mamele pentru simptomele copiilor ...

39971772-Autismul | Sandor Valentina - Academia.edu
https://www.academia.edu/8749695/39971772-Autismul
Este general acceptat faptul, că **autismul** este cauzat de anormalităţi în funcţionarea şi structurarea creierului, dar, nu se ştie încă, ce anume din funcţionarea şi ...

Ce este autismul? | Voinicel - Centrul de intervenție precoce
www.voinicel.md/index.php?pag=news&id=725&rid=477&l=ro
Termenul de "autism" este folosit în sensul larg de "tulburări din spectrul **autismului**". **Autismul** este o tulburare de dezvoltare, de natura neuro-biologica, ci nu ...

Invingem Autismul
www.invingemautismul.ro/
Cine suntem Asociatia Învingem **Autismul** a fost înfiintata în anul 2009, cu scopul de a îmbunatati viata persoanelor afectate de tulburari din spectrul **autismului**.

Autismul - Pro Autist Alapítvány
proautist.ro/ro/autismul
Despre autism. **Autismul** este o tulburare a dezvoltării individuale calitative și a dezvoltării abilităților de comunicare cognitive, care poate cauza o stare de ...

#autismul hashtag on Twitter
https://twitter.com/hashtag/autismul?src=hash
See Tweets about **#autismul** on Twitter. See what people are saying and join the conversation.

6 lucruri mai putin cunoscute despre tulburarile de spectru autist ...
https://www.reginamaria.ro/.../6-lucruri-mai-putin-cunoscute-despre-tulburarile-de-sp...
Tulburarile de spectru **autist** afecteaza copiii de orice etnie, rasa sau conditie socioeconomica, fiind de 4,5 ori mai frecvent intalnite in cazul baietilor decat la ...

Ce este Autismul? | SOS Autism
autismmoldova.md/ro/autism/ce-este-autismul
Autismul este o tulburare pervazivă (profundă) de dezvoltare de etiologie necunoscută, care afectează dezvoltarea normală a copilului, apare in primii 3 ani de ...

Şi copiii cu autism merg la şcoală - Asociaţia Învingem Autismul
https://www.fundatiaorange.ro/si-copiii-cu-autism-merg-la-scoala-asociatia-invingem-...
Şi copiii cu autism merg la școală - Asociația Învingem **Autismul**. Fundația Orange / Susține un ONG /. Şi copiii cu autism merg la școală - Asociația ...

autismul - Traducere în engleză - exemple în română | Reverso Context
https://context.reverso.net/traducere/romana-engleza/autismul
Reverso Context oferă traducere în context din română în engleză pentru "**autismul**", cu exemple: Chiar și atunci **autismul** era considerat o boală.

Copiii care au învins autismul - Partea I ! Povestea evoluției lui B ...
https://atca.ro › Articole
3 aug. 2017 - La început, totul părea în regulă cu băiețelul lui L. Atingea fiecare treaptă de dezvoltare și se bucura de fiecare descoperire pe care o făcea.

Autismul poate fi detectat cu mult timp înainte ca simptomele să apară ...
https://ziarulprahova.ro › Actualitate
7 iul. 2018 - Scanări ale creierului pot detecta **autismul** cu mult înainte de apariţia vreunui simptom, susţin oamenii de ştiinţă. În prezent, cea mai timpurie ...

(PDF) O ALTĂ FORMĂ DE AUTISM – AUTISMUL VIRTUAL
https://www.researchgate.net/.../323425282_O_ALTA_FORMA_DE_AUTISM_-_AUTI...
27 feb. 2018 - OBJECTIVES: Identifying the incidence of excessive use of virtual environment between 2012-2017 in children recently diagnosed with autism.

Autismul nu este marginea prapastiei - Liviu Predescu - eMAG.ro
https://www.emag.ro › ... › Medicina si farmacie › Medicina si farmacie Paralela 45
Cumpara **Autismul** nu este marginea prapastiei - Liviu Predescu de la eMAG! Descopera promotiile zilei, ai preturi avantajoase, livrare rapida, plata in rate, ...

8 afecțiuni care se pot asocia cu autismul - Autism, Terapie ...
https://autismvoice.ro › Articole
23 feb. 2018 - 8 afecțiuni care se pot asocia cu **autismul**. Sunt afecțiuni care nu apar singure și pot fi însoțite și de altele. Spre exemplu, persoanele cu ...

Terapeutul care a adus în România o metodă unică în tratarea ...
https://viitorulromaniei.ro/.../terapeutul-care-a-adus-in-romania-o-metoda-unica-in-tra...
31 oct. 2017 - **Autismul** este la el acasă în această lume, pentru că se schimbă pur și simplu genomul uman. Din anul 2000 și până în 2010, prevalența ...

"Vaccinul nu cauzează autism, pentru că nimeni nu știe ce cauzează ...
desprevaccin.ro/testimonial-maria-coman/
20 oct. 2016 - Nu, vaccinul nu cauzează autism, pentru că, din păcate, nimeni nu știe ce cauzează **autismul**. Da, e adevărat, unele cazuri de autism devin ...

Este autismul următoarea etapă evolutivă a omenirii? | psihoactiv
https://www.psihoactiv.com/.../este-autismul-urmatoarea-etapa-evolutiva-a-omenirii/
16 oct. 2018 - La întrebarea este **autismul** următorul stadiu al evoluției umane, o serie de specialiști în tulburările din spectrul **autismului** răspund cu ...

PressReader - Femeia: 2019-01-29 - Autismul, vedere din interior
https://www.pressreader.com/romania/femeia/20190129/281663961197741
29 ian. 2019 - Pare aproape o contradicţe în termeni, nu-i așa? Percepția prevalentă este că **autismul** e o lume închisă, iar comunicarea cu ea, aproape ...

Semnele care prevestesc autismul - Cronica Romana
https://cronicaromana.net › Sanatate
19 feb. 2018 - **Autismul** este o tulburare de dezvoltare considerata drept una dintre cele mai severe tulburari neuropsihiatrice ale copilariei.

Imagini pentru autismul

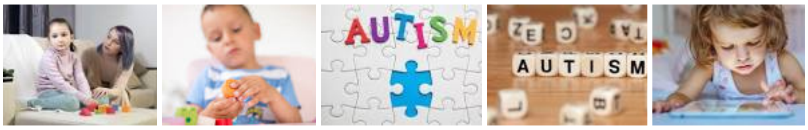

Mai multe imagini pentru autismul Raportați imagini

[PDF] Ce cauzează autismul? - Autism Speaks
https://www.autismspeaks.org/sites/default/files/100-day-kit-romanian.pdf
Copilul tău a fost diagnosticat cu autism sau cu o tulburare de spectru **autist** şi tu ai ... cunoscute şi ca tulburări de spectru **autist**, incluzând **autismul**, Tulburarea ...

[Autismul: fiul meu și blogul meu - Opus Dei](https://opusdei.org)
https://opusdei.org › Știri › Mărturii ▾
18 iul. 2013 - Viviana Miskulin are patru copii, unul dintre ei fiind **autist**. Femeia de origine peruviana a început acum 4 ani un blog: „Educando a mi hijo" ...

Autismul, tratat la negru - Jurnalul National - Antena3
https://jurnalul.antena3.ro/campaniile...autista/autismul-tratat-la-negru-137370.html ▾
"E **autist**, doamnă, nu e vina nimănui. Ce-i aia autism?! Adică la 20 de ani copilul dumneavoastră n-o să ştie care e stângul şi care e dreptul". Şi medicamente ...

Consumul de canabis ar putea trata autismul - VICE
https://www.vice.com/ro/article/.../consumul-de-canabis-ar-putea-trata-autismul-337 ▾
8 mar. 2016 - Am vorbit cu autoarea studiului, care spune că cercetătorii au abordat-o deja ca să afle cum poate fi folosit canabisul pentru tratarea **autismului**.

AUTISMUL SI SINDROMUL ASPERGER (Romanian Edition ...
https://www.amazon.com/AUTISMUL-SI-SINDROMUL-ASPERGER.../6065870595 ▾
AUTISMUL SI SINDROMUL ASPERGER (Romanian Edition) [Christopher Barber] on Amazon.com. *FREE* shipping on qualifying offers. Cartea isi propune sa ...

AUTISMUL SI TERAPIA CU CELULE STEM - Biohellenika
https://www.biohellenika.gr/el/profil/.../331-autismul-si-terapia-cu-celule-stem.html ▾
Autismul si bolile aferente lui apartinind aceleiasi categorii de autism , in ultimii ani apar ca o epidemie si baietii sunt cel mai des afectati . Desi diagnosticul ...

Daniela Bololoi, președinte Help Autism: „Autismul nu ţine cont de ...
https://intervio.ro › Societate Civilă ▾
22 iun. 2017 - Deşi incidenţa ingrijorătoare ar plasa-o fără îndoială în rândul problemelor de sănătate naţională, tulburarea de spectru **autist** primeşte încă ...

Opinii :: Viaţa cu autism. „Autismul nu e răceală să treacă. M-am ...
www.hotnews.ro/stiri-22839908-.htm?nomobile ▾
29 nov. 2018 - M-am săturat de toți cei care nu înțeleg **autismul**! M-am săturat, sunt paşnică de felul meu, dar toate au o limită. Îmi vine uneori să-mi inchid ...

Autismul Infantil. Structuri Psihopatologice Si Terapie Complexa ...
https://www.scribd.com/.../Autismul-Infantil-Structuri-Psihopatologice-Si-Terapie-Co... ▾
Evaluare: 5 - 5 voturi
AUTISMUL INFANTIL. STRUCTURI PSIHOPATOLOGICE I TERAPIE COMPLEX Cluj-Napoca, 2004 1. CUPRINS 1. **AUTISMUL** - UN MIT TENACE Scurt istoric ...

Autismul sau neputinţa de a trăi clipa | Doxologia
https://doxologia.ro/familie/terapie-pentru-suflet/autismul-neputinta-de-trai-clipa ▾
Autismul este o tulburare care provoacă drame cumplite în familiile afectate. Este o afecțiune care nu se ştie de unde vine şi cum se poate vindeca. Una dintre ...

Invatam cu Pictograme: „Autismul: O viata in culori" « Timotion
https://www.timotion.ro/ce.../invatam-cu-pictograme-autismul-o-viata-in-culori/ ▾
Proiectul **Autismul**: O Viață în Culori vine în sprijinul copiilor diagnosticați cu autism. Principalele arii afectate în autism sunt: comunicarea și limbajul, ...

Se fac bine copiii autisti cand se fac mari? - Interviu - Ziare.com
www.ziare.com › Scoala › invatamant special ▾
23 iun. 2014 - Auzim frecvent de autism si de copii **autisti**. Accesul la informatie a facut sa ne familiarizam cu fenomenul, desi contactul cu o astfel de ...

Atelier LiterNet › Arhiva rubricii Autismul, din interior
https://atelier.liternet.ro/arhivarubricii/130/Autismul-din-interior.html
2 mai 2011 - Lista tuturor articolelor publicate în rubrica **Autismul**, din interior.

Asociatia Invingem Autismul - Bursa Binelui
https://www.bursabinelui.ro/BursaBinelui/ONG-uri/Asociatia-Invingem-Autismul ▾
Asociația Invingem **Autismul** este o organizație neguvernamentală care are ca misiune să imbunătățească viața persoanelor afectate de tulburări din ...

Tânăra care demonstrează în fiecare zi că autismul nu este o boală ...
www.unitedautism.ro/.../tanara-care-demonstreaza-in-fiecare-zi-ca-autismul-nu-este-o-... ▾
Autismul nu este o boală și nu este contagiosa, iar persoanele cu autism sunt copii și tineri ca toți alții, cu dorințe și visuri, doar că necesită o abordare mai ...

A fost descoperita o cauza a autismului | Sound of Science
soundofscience.info › Medicina › A fost descoperita o cauza a autismului
A fost descoperita o cauza a **autismului**, o boala care i-a pus in dificultate pe oamenii de stiinta din toata lumea inca de la descoperirea sa in anii '40.

REPORTAJ Autismul, tulburarea copiilor tăcuţi | adevarul.ro
https://adevarul.ro › Sănătate › Medicină
6 ian. 2013 - Nu pot sta locului, rostesc tarziu primele cuvinte si comunica dificil. Sunt copii **autisti** si, desi cauzele sunt inca o necunoscuta, solutia este ca ...

6 lucruri despre autismul non-verbal – Știri pentru viață
https://stiripentruviata.ro › dizabilităţi › autism
2 dec. 2015 - Să fim sinceri, nu toată lumea este familiarizată cu **autismul**. Nu este **autismul** de tipul filmului „Rain Man", cu un personaj puțin ciudat, dar ...

Sa invingem autismul: Amazon.co.uk: Raun K. Kaufman ...
https://www.amazon.co.uk/Sa-invingem-autismul-Raun-Kaufman/dp/6067890194
Buy Sa invingem **autismul** by Raun K. Kaufman (ISBN: 9786067890198) from Amazon's Book Store. Everyday low prices and free delivery on eligible orders.

S-a descoperit de ce apare autismul și nu e vorba de vaccinuri
https://noizz.ro/stuff/s-a-descoperit-de-ce-apare-autismul-si-nu-e...de.../hwsl89r
21 sept. 2017 - În urmă cu o săptămână a fost publicat un studiu științific care indică cauza principală pentru apariția **autismului** la copii. Vaccinurile și ...

Broccoli ar putea reprezenta secretul pentru tratarea autismului - Rador
www.rador.ro › inedit
16 oct. 2014 - Este posibilă crearea primei pilule care să influenţeze **autismul** încă de la declanşarea bolii. Medicamente existente doar controlează ...

[PDF] Autismul Comunicarea - PECS
www.pecs-romania.com/PECSFlyerRomanian2012.pdf
Autismul şi. Comunicarea. Copilul tău a fost diagnosticat cu autism sau alta disabilitate de dezvoltare? Există diverse opţiuni de tratament şi terapii disponibile.

Liviu Predescu - Cu autismul la psiholog - - elefant.ro
www.elefant.ro/carti/carte/self-help/...si.../cu-autismul-la-psiholog-208571.html
Cu **autismul** la psiholog - Liviu Predescu - - Aceasta carte, publicata la editura For you, va schimba perceptia asupra **autismului**. Ea este dedicata tuturor pari.

Autismul - KFetele
https://www.kfetele.ro/tags/autismul/
Lifestyle. Ce sanse reale de recuperare au copiii cu autism in Romania! Anca Dumitrescu, primul specialist roman acreditat international in terapia ABA, ...

Tulburarile de Spectru Autist (autism) - Centrul Medical Bellanima
www.bellanima.ro/tulburarile-de-spectru-autist-autism/
Tulburarile de spectru **autist** (autism) reprezinta un grup de tulburări de dezvoltare a copilului caracterizate printr-o deteriorare calitativă, severă şi pervazivă in ...

Invingem Autismul: Centru multidisciplinar Învingem Autismul
donatie.ro/index.../invingem-autismul-centru-multidisciplinar-invingem-autismul
Invingem **Autismul**: Centru multidisciplinar Învingem **Autismul**. Print; Email. Pentru ca a fi diferit nu inseamna a fi exclus. Pentru ca fiecare copil este valoros si ...

Antidepresivele și autismul - SciNews
https://scinews.ro/antidepresivele-si-autismul/
14 dec. 2015 - Cauzele **autismului** sunt complexe și încă neînțelese complet. Cea mai bună explicație de până acum, reconfirmată recent de o nouă ...

Stop autismului virtual - InfoCons
https://www.infocons.ro/ro/pg-stop-autismului-virtual-MjI4LTEtMTM2.html
Stop **autismului** virtual! Adresează-te specialistului dacă copilul tău la un an și șase luni NU are următoarele achiziții: Nu arată cu degetul; Nu răspunde la ...

Cum recunoastem autismul – Tot ce trebuie sa stii
https://www.tcts.ro/cum-recunoastem-autismul/
Trăsături caracteristice ale copilului **autist**. – incapacitatea de a adopta o poziţie normală în timp ce este luat în braţe. Dacă debutul bolii este precoce, spre luna ...

[Autismul: teorie și intervenție educațională by Theo Peeters](https://www.goodreads.com/book/show/29069915-autismul)
https://www.goodreads.com/book/show/29069915-autismul ▼
Evaluare: 3,4 - 11 voturi
Autismul book. Read reviews from world's largest community for readers. Persoanele care sufera de autism se confrunta cu probleme atunci cind trebuie sa ...

Autismul - Între mit și realitate - Facebook
https://www.facebook.com/events/2140659119554922/ ▼
Autismul - Între mit și realitate. Public. · Hosted by Editura Herald. Interested. clock. Monday, April 2, 2018 at 6:30 PM – 8:00 PM UTC+03. about 12 months ago ...

“Autismul virtual”, afecțiunea de care suferă copiii lăsați excesiv în ...
https://mediastandard.ro/autismul-virtual-afectiunea-de-care-sufera-copiii-lasati-excesi... ▼
Jocurile pe telefon sau desenele animate par inofensive, dar pot avea efecte grave pentru copii, mai ales dacă sunt mai mici de doi ani. În urma unor cercetări ...

Doi cercetători au descoperit de ce apare autismul - Digi24
https://www.digi24.ro/stiri/.../doi-cercetatori-au-descoperit-de-ce-apare-autismul-7968... ▼
20 sept. 2017 - Cei doi au descoperit că prezența unei anumite bacterii în sistemul digestiv al mamei poate duce la **autismul** copilului. Pe lângă asta ...

Cărți - Editura Polirom
https://www.polirom.ro/carti/-/carte/5975 ▼
Autismul. Teorie si interventie educationala este un studiu de referinta pentru toti specialistii care lucreaza cu copilul sau adultul cu autism – medici, psihologi, ...

Ce este Autismul? on Vimeo
https://vimeo.com › People CD Inc. › Videos

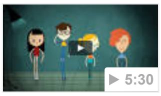

30 iun. 2018 - Încărcat de People CD Inc.
O introducere în autism care urmărește să sensibilizeze publicul tânăr care nu are autism, să stimuleze ...

Autismul, în România. Peste 30 000 de copii suferă de această boală ...
https://www.realitatea.net/autismul-in-romania-peste-30-000-de-copii-sufera-de-aceast... ▼
2 apr. 2017 - comentarii, Astazi este Ziua Internaţională a **Autismului**. Ocazie cu care s-au organizat manifestări în întreaga lume. La Bucureşti, de exemplu, ...

De acum, autismul la copii poate fi diagnosticat de la un an. Află cum!
www.monitorulcj.ro/.../27035-da-acum-autismul-la-copii-poate-fi-diagnosticat-de-la-... ▼
12 aug. 2013 - Cercetători americani au anunţat că au dezvoltat un nou un mijloc de a diagnosticarea **autismului** la copii de la vârsta de un an.

Autismul La Prescolari - Margareta Gifei - Libris
https://www.libris.ro › Carti › Stiinte Umaniste ▼
Autismul pare sa fie prezent peste tot in jurul nostru in ultima vreme in ziare, la TV, chiar si pe afise. Informatiile arata ca rata prezentei sale este in crestere ...

Semne care trădează autismul la copii - Copii - MAMA.md - все о ...
https://mama.md › Новости | Noutati › Copii ▼
Autismul este o afecţiune considerată drept una dintre cele mai frecvente tulburări ale copilăriei, mai frecventă decât cancerul, diabetul şi sindromul Down.

Peste 30.000 de copii diagnosticați cu autism, în România. Simptome ...
https://a1.ro/.../autismul-ce-este-autismul-simptome-factori-de-risc-si-tratament-id8464... ▼
2 apr. 2019 - **Autismul**. Ce este **autismul**, simptome, factori de risc și tratament. Află despre tulburările de spectru **autist**, care sunt factorii de risc și cum pot fi ...

Autismul - The Titi Tudorancea Library
https://www.tititudorancea.org/z/autismul.htm ▼
Ce este **autismul**? Care sunt cauzele **autismului**? Cand se manifesta de obicei semnele **autismului**? Care sunt unele dintre posibilele semne de autism?

Autismul - o provocare pentru medicii moldoveni - Radio Europa Liberă
https://moldova.europalibera.org/a/24945987.html ▼
2 apr. 2013 - În lume astăzi este ziua pentru conştientizarea **autismului**, o denumire generic pentru o serie de tulburări comportamentale fundamentale.

Ziua Internațională de Conștientizare a Autismului - USR pledează ...
https://www.usr.ro/2019/04/02/ziua-internationala-de-constientizare-autismului/ ▼

2 apr. 2019 - Pe 2 aprilie celebrăm Ziua Internațională de Conștientizare a **Autismului**. **Autismul** este o tulburare de dezvoltare care presupune dificultăţi în ...

Autismul, din perspectiva unui băiat de zece ani - Supereroi printre noi
supereroiprintrenoi.ro/interviu/interviu-baiat-autism/
David este un băiat de zece ani, **autist**. Are doi frați mai mici, deasemenea cu autism, merge la școală iar în timpul liber citește sau merge la cinema.

Autismul este o boala genetica - Personal Genetics
https://personalgenetics.ro/articol-pacienti/autismul-este-o-boala-genetica/
1 din 68 de copii sunt diagnosticati cu autism conform CDC's Autism and Development Disabilities Monitoring (ADDM). – **Autismul** apare in toate tipurile de rase ...

[PDF] Autismul nu dispare la 18 ani! - Pentru Voi
www.pentruvoi.ro/uploads/files/2009_Autismul_nu_dispare_la_18_ani.pdf
copiilor cu autism şi a lipsei serviciilor de suport specializate pentru adulţii cu autism. **Autismul** nu este considerat încă în România ca o dizabilitate de dezvoltare ...

Autismul, discriminarea și discernămîntul | Contributors
www.contributors.ro › Opinie › Societate/Life
20 feb. 2018 - În orice fel ai întoarce-o semantic, **autismul** nu poate fi asociat cu dezinformarea și cu faptul de a cunoaște realitatea. Redau și eu o parte din ...

[PDF] Anul 2019 „Să înţelegem autismul! Şanse egale şi participare activă!”
www.dspb.ro/diverse/dspb/2019/210319/Pliant%20parinti.pdf
Folosiţi resursa TIMP pentru diagnosticarea precoce a **autismului**! • Informaţi-vă asupra comportamentelor specifice copilului cu autism prin participare.

Un studiu nou privind uleiul CBD și autismul susține bănuielile ...
https://ulei-cbd.ro/blog/studiu-nou-privind-uleiul-cbd-si-autismul
3 feb. 2019 - Studiul "Experiența în viața reală în cazul tratării **autismului** cu ulei CBD. Analiza siguranței și eficacității" a urmărit 188 de pacienți cu ...

Pharma Business | Autismul afecteaza intregul creier - Pharma Business
https://www.pharma-business.ro/autismul-afecteaza-intregul-creier/
15 oct. 2006 - Un studiu recent realizat de neurologi si psihiatrii de la University of Pittsburgh School of Medicine a scos la iveala faptul ca **autismul** este o ...

Boli psihice //facts - Autismul - Wattpad
https://www.wattpad.com/559541667-boli-psihice-facts-autismul
Read **Autismul** from the story Boli psihice //facts by AntoniaPetrut1 (Bulletproof <3) with 4 reads. bolipsihice. **Autismul** este o tublurare de dezvoltare (malfo...

Autismul-consecințele întârzierii tratamentului - NewsMed.ro
https://newsmed.ro/autismul-imactul-terapiilor-timpurii/
Autismul este tulburarea neuropsihiatrică principală din cadrul tulburărilor din spectrul **autist**. Această tulburare afectează din ce în ce mai mulţi copii,

Autismul — cum să depăşim dificultăţile unei boli ciudate ...
https://wol.jw.org/ro/wol/d/r34/lp-m/101995089
Autismul — cum să depăşim dificultăţile unei boli ciudate. CHRISTOPHER era un băieţel drăguţ, cu un comportament normal, care, de la vârsta de un an şi şase ...

Autismul în Teleorman, gestionat mai ușor cu fonduri europene ...
https://presshub.ro/proiecte.../2019/.../autism-teleorman-fonduri-europene-bihor-alba/
4 feb. 2019 - presshub.ro își finanțează întregul conținut jurnalistic prin publicitate. Partenerii noștri folosesc tehnologii precum cookie-urile de pe ...

Ce este Autismul? – Autism Romania
https://autismromania.ro/despre-autism/ce-este-autismul/
Autismul este o tulburare complexă de origine neurobiologică ce afectează abilitățile de comunicare și relaționare ale persoanei cu cei din jur, modul în care ...

DESPRE AUTISM LA COPII | Parenting Romania
www.parentingromania.ro/despre-autism-la-copii/
Autismul este o tulburare severă și pervazivă care afectează toate ariile de dezvoltare ale copilului, nu se vindecă niciodată dar poate fi ameliorată semnificativ ...

Autismul, detectat prin analize de sânge | Ziarul Cuget Liber de ...
https://www.cugetliber.ro/redirect-stiri-sanatate-autismul-detectat-prin-analize-de-sang...

8 ian. 2013 - **Autismul** ar putea fi depistat încă din primele zile de viaţă ale copilului, pentru ca acesta să nu mai treacă prin suferinţa cruntă de mai târziu, ...

Informare şi conştientizare asupra autismului - Copiii de Cristal
https://www.copiiidecristal.ro/informare-si-constientizare-asupra-autismului/
Ziua Internațională a **Autismului**. Anual, cea mai importantă campanie de informare și conștientizare organizată de Asociaţia „Copiii de Cristal" este de Ziua ...

2 aprilie - Ziua Internațională de Conștientizare a Autismului - Timlogo
https://timlogo.ro/noutati/.../2-aprilie-ziua-internationala-de-constientizare-a-autismulu...
2 apr. 2019 - În 2008, Adunarea Generală a Națiunilor Unite a declarat în unanimitate 2 aprilie ca Ziua Mondială a **Autismului** pentru a evidenția nevoia de ...

Ziua Internațională de Conștientizare a problemelor Autismului
https://msmps.gov.md/.../2-aprilie-ziua-internationala-de-constientizare-problemelor-a...
Anual, la data de 2 aprilie, la nivel mondial, este marcată Ziua Internaţională de conştientizare a **autismului**, cu scopul de a dinamiza eforturile de promovare a ...

Autismul nu doare, indiferența da! - TRINITAS TV
https://www.trinitas.tv › Emisiuni › Sociale › Secvențe Trinitas
8 apr. 2019 - Prin intermediul TRINITAS TV, o televiziune cultural-religioasa, Patriarhia Română informează opinia publică despre evenimentele bisericeşti, ...

Să învingem autismul. Metoda revoluționară care a ajutat familii din ...
https://books.google.ro/books?isbn=6067890615
Raun K. Kaufman - 2017 - Psychology
Crede-mă când îți spun că, dacă ai un medic care chiar este bine informat în privința **autismului**, nu vei avea astfel de experiențe. Un alt factor important pe ...

Autismul virtual și efectele sale asupra evoluției copilului | Revista ...
https://iteach.ro/.../autismul-virtual-si-efectele-sale-asupra-evolutiei-copilului
2 iul. 2018 - **Autismul** virtual este una dintre enigmele care, de ceva timp, au devenit fie marea provocare, fie marea necunoscută, dar, în același timp, și ...

Ce este autismul ? – Editura Trei Blog
blog.edituratrei.ro/ce-este-autismul/
28 mar. 2013 - ... cu ocazia Zilei Internationale de Constientizare a **Autismului** – 2 aprilie. ... Sorescu – parinte, Director executiv – Asociatia Invingem **Autismul**

Ce este autismul şi care sunt miturile care se vehiculează în legătură ...
www.radiocluj.ro/.../ce-este-autismul-si-care-sunt-miturile-care-se-vehiculeaza-in-legat...
2 apr. 2019 - Ce este **autismul** şi care sunt miturile care se vehiculează în legătură cu această tulburare de dezvoltare? Cât de periculoasă este capcana ...

Carte despre autism Neurotriburi. Istoria uitata a autismului - Steve ...
https://editurafrontiera.ro/.../steve-silberman-neurotriburi-istoria-uitata-a-autismului/
Carte despre autism Neurotriburi de Steve Silberman. **Autismul** are o istorie fascinanta, plina de experimente indraznete, pasiuni solitare si impostura. Cartea ...

Căutări referitoare la autismul

autismul **definitie**
autismul **referat**
autismul **cauze**
evolutia autismului
autismul **se vindeca**
autismul **virtual**
autismul **la adulti**
tratamentul autismului

1 2 3 Înainte

România | **Comuna Beica de Jos** - Pe baza activităților anterioare - Utilizați locația exactă - Aflați mai multe

Ajutor Trimiteți feedback Confidențialitate Termeni
