# Supplementary material for: Autism-Related Information on Websites and General-Purpose Artificial Intelligence Chatbots: Comparative, Bilingual Study
Source: JMIR Form Res. 2026 Jul 13;10:e85196. doi: 10.2196/85196 (PMC13361620; doi:10.2196/85196)
Supplement: Multimedia Appendix 3 [file formative-v10-e85196-s003.docx]

**CHECKLIST FOR ASSESSING THE QUALITY OF AUTISM SPECTRUM DISORDER–RELATED INFORMATION PROVIDED BY WEBSITES AND CHATBOTS (EN)**

| **I. Sections** | **II. Items to be assessed** |
| --- | --- |
| Definition and epidemiology (What is autism, and how prevalent is it?) | 1. Autism spectrum disorder (ASD) is a complex developmental condition that involves persistent difficulties in social interaction, verbal and nonverbal communication, and restricted and/or repetitive patterns of behavior. 2. The diagnosis of ASD includes several conditions that, before the introduction of DSM-5, were diagnosed separately (autistic disorder, Asperger syndrome, childhood disintegrative disorder, Rett syndrome, and pervasive developmental disorder not otherwise specified). 3. Most children with ASD are diagnosed in childhood, at around 2-3 years of age, based on the most obvious signs. Some children with autism appear to develop normally until childhood, when they stop acquiring new skills or lose previously acquired skills. 4. It is estimated that worldwide 1 in 160 children has autism. ASD is three to four times more common in boys than in girls, and many girls with ASD show less obvious signs than boys. Autism is a lifelong disorder. |
| Symptoms (How does it manifest, and how can it be recognized?) | 5. Symptoms can vary in intensity (from mild to severe) and often change over time. Manifestations differ from person to person. |
|  | The features of autism spectrum disorder fall into two categories: 6. Problems with social interaction and communication: difficulties in conversation, reduced expression of emotions, difficulty understanding or responding to social cues such as eye contact and facial expressions, and deficits in developing / maintaining / understanding relationships with others. 7. Repetitive patterns of behavior, unusual interests or activities, speaking in an unusual manner, a marked need for routine or predictable structure, intense interest in activities unusual for a child of a similar age, experiencing sensory aspects of the world in unusual or extreme ways (E.g. pain / temperature, excessive smelling / touching of objects, fascination with lights and movement, being overwhelmed by loud noises, etc.), and others. |
|  | 8. Many people with autism have normal intelligence, while others have mild or significant intellectual delay (about one third develop some form of intellectual developmental delay). 9. Other symptoms include hyperactivity, impulsivity, aggression, attention-focusing difficulties, unusual emotional reactions, and unusual eating and sleeping habits. |
|  | 10. Early diagnosis and treatment are important for reducing autism symptoms and improving the quality of life of people with autism and their families. 11. There is no specific medical test for autism. It is diagnosed by observing how the child speaks and behaves compared with other children of the same age. Specialists usually diagnose autism by talking with the child and asking questions to parents and other caregivers. |
| Risk factors | 12. The exact causes of ASD are unknown. 13. Several factors probably contribute to autism: - genetic conditions - Down syndrome, fragile X syndrome, Rett syndrome - older parents - very low birth weight - siblings with ASD - environmental factors 14. Research has shown that it is not caused by deficiencies in the parent-child relationship or by vaccines. |
| Course and complications | 15. Although ASD is generally not cured, studies have shown that symptoms can improve with early diagnosis and treatment. In some children with ASD, the first signs of future problems appear in the first months of life. In others, symptoms may not appear until 24 months of age or later. |
|  | 16. Patients with ASD are at higher risk for certain medical conditions, such as sleep problems, learning difficulties, epilepsy, dyslexia, dyspraxia, and mental disorders. |
| Treatment | 17. There is no single treatment for autism. 18. Treatments may include intensive educational skill-building and teaching sessions, known as applied behavior analysis (ABA), as well as many other interactive, child-centered approaches. ABA helps individuals learn social, motor, and verbal behaviors, as well as reasoning skills. It is based on teaching skills through observation and positive reinforcement. 19. Treatment may also include special training and support for parents, speech and language therapy, occupational therapy, and/or social skills training. 20. Some children and adults with ASD experience psychological problems such as anxiety, ADHD, disruptive behaviors, or depression. These difficulties may be treated with psychotherapy or medication. |
|  | 21. Close collaboration with a doctor or health professional is an important part of finding the right treatment plan. |
|  | 22. Play therapy can help people with ASD improve their social and emotional skills, think in different ways, improve their language or communication skills, and expand the ways in which they play with toys. |
|  | 23. Occupational therapy can help people with ASD gain knowledge and develop skills for independent living. Occupational therapists work as part of a team that includes parents, teachers, and other specialists. |
| Alternative treatments | 24. Always talk to a doctor if there is anything you are unsure about. Some false treatments can be very dangerous, such as: - special diets - gluten-free, casein-free, or ketogenic - vitamins, minerals, and food supplements - GcMAF - an injection made from blood cells - memory medications - hyperbaric oxygen therapy. |

**Explanations regarding content evaluation**

**Completeness score (CS)**

- Shows how complete the presentation of the disease/topic is.
- The evaluation is performed using as reference the numbered information (E.g. 1., 2.) in column II of the benchmark table (Items to be assessed).
- The numbered information in column II is searched for in the text saved from the website, and **1 point** is awarded for each item found (regardless of how correctly or incorrectly it was presented, and regardless of how briefly or in how much detail it was presented).
- Information that is completely omitted (not mentioned even briefly) is scored **0 points**.
- The score is entered in the data.xls file, in the cell corresponding to the evaluated website, in the column for the CS score of the evaluated information (E.g. C1).

**Accuracy score (AS)**

- Shows how correct the presented information is.
- The correctness of the information is checked using, again, the numbered information in column II of the table as the reference.
- **2 points** are awarded if the information is fully correct (minor inaccuracies may be accepted).
- **1 point** is awarded if the correctness of the information is acceptable (satisfactory).
- **0 points** are awarded if the information is incorrect (or presented with serious, unacceptable inaccuracies).
- The score is entered in the data.xls file, in the cell corresponding to the evaluated website, in the column for the AS score of the evaluated information (E.g. A1).
- If a certain numbered item was not presented at all (CS=0 was recorded for the completeness score), NA (meaning “Not applicable”) is entered in the AS cell corresponding to the missing information.

**Information with potential risk (through its presence or absence on the website)**

- Users could be exposed to risks regarding their health status or even their lives, either by applying incorrect recommendations found on the website or by the absence (omission) of essential information.
- If such information/recommendations are identified on any of the evaluated websites, they must be copied and pasted into the data.xls file, in the column marked in red. (The sentence or paragraph in which the information appears should be copied.)

**BAREM DE EVALUARE A CALITĂȚII INFORMAȚIILOR DESPRE TULBURAREA DE SPECTRU AUTIST FURNIZATE DE WEBSITE-URI ȘI CHATBOTURI (RO)**

| **I. Capitole** | **II. Itemi de evaluat** |
| --- | --- |
| Definiţie şi epidemiologie (Ce se înţelege prin depresie şi cât de răspândită este?) | 1.Tulburarea de spectru autist (TSA) este o afecțiune complexă de dezvoltare care implică dificultăți persistente în interacțiunile sociale, comunicarea verbală și non-verbală și tipare de comportament cu caracter restrâns și/sau repetitiv.  2. Diagnosticul de TSA include mai multe afecțiuni care înainte de aparitia DSM V erau diagnosticate separat (tulburarea autistă, tulburarea Asperger, tulburarea dezintegrativă a copilăriei, sindromul Rett, tulburarea pervazivă de dezvoltare nespecificată în alt mod).  3. Cei mai mulți copii cu TSA sunt diagnosticați în copilărie la vârsta de 2-3 ani pe baza celor mai evidente semne. Unii copii cu autism se dezvoltă normal până la vârsta copilăriei, când încetează să mai dezvolte abilități noi sau pierd abilitățile dobândite anterior.  4. Se estimează că la nivel mondial 1 copil din 160 are autism. TSA este de trei până la patru ori mai frecventă la băieți decât la fete, iar multe fete cu TSA prezintă semne mai puțin evidente în comparație cu băieții. Autismul este o tulburare care durează pe tot parcursul vieții. |
| Simptome (Cum se manifestă, după ce o recunoaştem?) | 5. Simptomele pot varia în intensitate (de la forme ușoare până la severe) și se schimbă adesea în timp. Simptomatologia diferă de la persoană la persoană. |
|  | Caracteristicile tulburării spectrului autism se încadrează în două categorii:  6. Probleme de interacțiune și comunicare socială: dificultăți în conversație, exprimarea redusă a emoțiilor, înțelegerea sau răspunsul la indicii sociale precum contactul vizual și expresiile faciale, deficite în dezvoltarea / menținerea / înțelegerea relațiilor cu ceilalți.  7. Tipare repetitive de comportament, interese sau activități neobișnuite, vorbirea într-un mod unic, o nevoie semnificativă pentru o rutină sau o structură previzibilă, prezintă interes intens față de activități neobișnuite pentru un copil de vârstă similară, experimentează aspectele senzoriale ale lumii într-un mod neobișnuit sau extrem (ex., durere / temperatură, miros excesiv / atingere de obiecte, fascinație pentru lumini și mișcare, fiind copleșit de zgomote puternice, etc.) și altele. |
|  | 8. Multe persoane cu autism au inteligență normală, altele au întârzieri intelectuale ușoare sau semnificative (o treime dezvoltă o formă de întârziere a dezvoltării intelectuale).  9. Alte simptome: hiperactivitate, impulsivitate, agresivitate, dificultate tulburări de focalizare a atenției, reacții emoționale neobișnuite, obiceiuri neobișnuite de mâncare și somn. |
|  | 10. Diagnosticul și tratamentul precoce sunt importante pentru reducerea simptomelor autismului și îmbunătățirea calității vieții pentru persoanele cu autism și familiile lor.  11. Nu există un test medical specific pentru autism. Este diagnosticat pe baza observării modului în care copilul vorbește și acționează în comparație cu alți copii de aceeași vârstă. De obicei, specialiștii diagnostichează autismul vorbind cu copilul și punând întrebări părinților și altor îngrijitori. |
| Factori de risc. | 12. Cauzele precise ale TSA sunt necunoscute.  13. Mai mulți factori contribuie probabil la autism:   - afecțiuni genetice- sindromul Down, sindromul X fragil, sindromul Rett - părinți în vârstă - greutatea la naștere foarte mică - frați cu TSA - factori de mediu   14. Cercetările au arătat că nu este cauzată de deficiențele în relația părinți-copii sau de vaccinuri. |
| Evoluţie şi complicaţii | 15. Deși, de regulă, TSA nu se vindecă, studiile au arătat că simptomele se pot îmbunătăți odată cu diagnosticul și tratamentul precoce. La unii copii cu TSA primele indicii ale viitoarelor problemele apar în primele luni de viață. La alții, simptomele pot să nu apară decât după 24 de luni sau mai târziu. |
|  | 16. Pacienții cu TSA prezintă un risc mai mare pentru unele afecțiuni medicale, cum ar fi probleme de somn, dificultăți de învățare, epilepsie, dislexie, dispraxie și boli mintale. |
| Tratament | 17. Nu există un singur tratament pentru autism.  18. Tratamentele pot include sesiuni educaționale intensive de formare a abilităților și predare, cunoscute sub numele de analiza comportamentală aplicată (applied behavior analysis - ABA) și multe alte versiuni interactive, centrate pe copii. ABA îi ajută să învețe comportamente sociale, motorii și verbale, precum și abilități de raționament. Se bazează pe predarea abilităților prin observare și consolidare pozitivă.  19. Tratamentul poate implica, de asemenea, o pregătire specială și sprijin pentru părinți, terapie vorbirii și limbajului, terapie ocupațională și / sau formare de abilități sociale.  20. Unii copii și adulți cu TSA au probleme psihologice cum ar fi anxietatea, ADHD, comportamente perturbatoare sau depresie. Aceste dificultăți pot fi tratate cu psihoterapie sau cu medicamente. |
|  | 21. Colaborarea strânsă cu un medic sau un profesionist din domeniul sănătății este o parte importantă în găsirea planului de tratament potrivit. |
|  | 22. Terapia prin joacă poate ajuta persoanele cu TSA să-și îmbunătățească abilitățile sociale și emoționale, să gândească în moduri diferite, să-și amelioreze abilitățile de limbaj sau de comunicare și să-și extindă modalitățile în care se joacă cu jucăriile. |
|  | 23.Terapia ocupațională poate ajuta persoanele cu TSA să obțină cunoștințe și sa-și dezvolte abilități pentru traiul independent. Terapeuții ocupaționali lucrează ca parte a unei echipe care include părinți, profesori și alți specialiști. |
| Tratamente alternative | 24. Discutați întotdeauna cu un medic dacă există ceva despre care nu sunteți sigur. Unele tratamente false pot fi foarte periculoase, precum  -diete speciale- fără gluten, cazeină sau ketogene.  -vitamine, minerale și suplimente alimentare  -GcMAF- injecție făcută din celule sanguine  -medicamente pentru memorie  -oxigenoterapie hiperbarică. |

**Explicații privind evaluarea conținutului**

**Scorul de exhaustivitate (SE)**

- Arată cât de completă este prezentarea bolii/subiectului.
- Evaluarea se face luând ca reper informațiile numerotate (ex. 1., 2.) din coloana II a tabelului-etalon (Itemi de evaluat**)**.
- Se caută în textul salvat de pe site informațiile numerotate din coloana II şi se acordă câte **1 punct** pentru fiecare informație găsită (indiferent cât de corect sau incorect a fost prezentată, indiferent cât de sumar sau detaliat a fost prezentată).
- Informațiile omise cu totul (nemenționate nici măcar sumar) se notează cu **0 puncte**.
- Punctajul se introduce în fișierul date.xls, în celula corespunzătoare site-ului evaluat, pe coloana scorului SE al informației evaluate. (ex.: E1)

**Scorul de acuratețe (SA)**

- Arată cât de corecte sunt informațiile prezentate.
- Se verifică corectitudinea informaţiilor luând ca reper (tot) informațiile numerotate din coloana II a tabelului.
- Se acordă **2 puncte** dacă corectitudinea informației este totală (se pot accepta inexactități neglijabile).
- Se acordă **1 punct** dacă corectitudinea informației este acceptabilă (satisfăcătoare).
- Se acordă **0 puncte** dacă informația este eronată (sau este prezentată cu inexactități serioase, inacceptabile).
- Punctajul se introduce în fișierul date.xls, în celula corespunzătoare site-ului evaluat, pe coloana scorului SA al informației evaluate. (ex.: A 1.)
- Dacă o anumită informație numerotată nu a fost prezentată deloc (la scorul SE s-a notat SE=0), în celula SA corespunzătoare informației lipsă, se introduce NA (cu semnificaţia „Neaplicabil”)

**Informaţii cu risc potențial (prin prezența sau absența lor pe website)**

- Utilizatorii ar putea fi expuși la riscuri în ce priveşte starea de sănătate sau chiar viata, fie prin punerea în aplicare a unor recomandări eronate găsite pe site, fie prin absența (omiterea) unor informaţii esențiale .
- În cazul în care pe oricare dintre site-urile evaluate se identifică astfel de informaţii/recomandări, acestea trebuie preluate prin Copy-Paste în fişierul date.xls, în coloana marcată cu roşu. (Se copiază propoziția sau paragraful în care apare informația).
