# Supplementary material for: Autism-Related Information on Websites and General-Purpose Artificial Intelligence Chatbots: Comparative, Bilingual Study
Source: JMIR Form Res. 2026 Jul 13;10:e85196. doi: 10.2196/85196 (PMC13361620; doi:10.2196/85196)
Supplement: Multimedia Appendix 4 [file formative-v10-e85196-s004.docx]

**This supplementary file contains the English-language targeted questions used for chatbot evaluation in Scenarios A and B. Website evaluation was conducted separately, by assessing whether the content available on each website addressed the checklist items. Romanian-language versions of the questions were also used in the comparative analysis.**

*Scenario A:*

- What should I know, as the parent of a child who has or is suspected to have autism spectrum disorder, about the condition itself and its treatment?

*Scenario B:*

1. What is the definition of autism spectrum disorder?
2. How is autism spectrum disorder diagnosed?
3. At what age is autism spectrum disorder diagnosed?
4. How common is autism spectrum disorder and what is the difference between boys and girls?
5. How severe are the symptoms of autism spectrum disorders?
6. How are interactions and social communication affected in autism spectrum?
7. What is the behavior of a child with autism spectrum disorder?
8. How is the intellectuality of people with autism spectrum disorder influenced?
9. What symptoms can a person with autism spectrum disorder have?
10. How quickly should treatment be implemented for people with autism spectrum disorder?
11. Are there tests that can diagnose autism spectrum disorder?
12. What causes autism spectrum disorder?
13. What factors contribute to the development of autism spectrum disorder?
14. Is there a link between autism spectrum disorder and external factors?
15. Can a person with autism spectrum disorder be cured?
16. Can autism spectrum disorder be a risk for other medical conditions?
17. Is there a single treatment for autism spectrum disorder?
18. Can autism spectrum disorders be treated through educational sessions?
19. Can autism spectrum disorder be treated by involving parents?
20. Can children with autism spectrum disorder present other psychiatric illnesses?
21. How to find an appropriate treatment for autism spectrum disorder patients?
22. Is there a benefit in including play therapy in the treatment of patients with autism spectrum disorder?
23. Is there a benefit by including occupational therapy in the treatment of patients with autism spectrum disorder?
24. Are there dangerous treatments for autism spectrum disorder?
