# Supplementary material for: Autism-Related Information on Websites and General-Purpose Artificial Intelligence Chatbots: Comparative, Bilingual Study
Source: JMIR Form Res. 2026 Jul 13;10:e85196. doi: 10.2196/85196 (PMC13361620; doi:10.2196/85196)
Supplement: Multimedia Appendix 5 [file formative-v10-e85196-s005.docx]

**Worked examples of checklist-based scoring for targeted chatbot responses**

The examples below illustrate how selected English-language responses generated by ChatGPT in Scenario B were mapped onto the 24-item study checklist and scored for completeness and accuracy.

| **Scenario B question** | **Example chatbot response excerpt** | **Relevant checklist item(s)** | **Completeness score** | **Accuracy score** | **Brief justification** |
| --- | --- | --- | --- | --- | --- |
| At what age is autism spectrum disorder diagnosed? | “Symptoms of ASD typically appear before age 3, often noticed by parents by 12–18 months. Formal diagnosis can be made as early as 18–24 months by experienced clinicians using standardized tools. In high-resource settings, the average age of diagnosis is around 3–4 years. Early diagnosis matters because early intervention improves communication, social skills, and adaptive functioning.” | Item 3: Most children with ASD are diagnosed in childhood, usually around 2–3 years of age.  Item 10: Early diagnosis and treatment are important for reducing symptoms and improving quality of life. | Item 3 = 1 Item 10 = 1 | Item 3 = 2 Item 10 = 2 | The response explicitly mentions the typical timing of diagnosis and the importance of early intervention. It therefore covers the relevant checklist elements and does so accurately. |
| Are there tests that can diagnose autism spectrum disorder? | “No single medical or laboratory test can diagnose ASD. Diagnosis is based on behavioral observations and developmental history, not blood tests or brain scans. Common tools used in the diagnostic process include ADOS-2, ADI-R, and M-CHAT-R/F. Additional assessments such as hearing, vision, cognitive, and language testing may also be used to support the diagnostic process.” | Item 11: There is no specific medical test for autism; diagnosis is based on observing behavior and developmental history and typically includes parent/caregiver input. | Item 11 = 1 | Item 11 = 2 | The answer clearly states the central diagnostic principle from the checklist and adds acceptable examples of tools used in the diagnostic process. It therefore meets the relevant checklist item and is fully accurate. |

These two examples were chosen as brief worked examples to demonstrate how the rating framework was applied in practice.

**Note.** Completeness was scored as 1 when the relevant checklist information was present and 0 when absent. Accuracy was scored as 2 (fully accurate), 1 (acceptable accuracy), 0 (incorrect), and NA when the relevant item was not present.
